# Enhancing the yield, fruiting body traits, and nutritional properties of five major edible fungi through the exploitation of ginger straw substrate

**DOI:** 10.3389/fnut.2025.1583716

**Published:** 2025-05-16

**Authors:** Yan Zhang, Yihui Wang, Yuting Li, Panmeng Wang, Li Wang, Zhuang Li

**Affiliations:** Shandong Provincial Key Laboratory of Agricultural Microbiology, College of Plant Protection, Taian, China

**Keywords:** ginger straw, edible fungi, yield, fruiting body traits, nutritional properties

## Abstract

Edible fungi, as nutritious foods in healthy diets, have gained popularity among consumers. The expansion of the edible fungi cultivation scale led to a shortage of cultivation substrate, making the development and utilization of new substrates a research hotspot. Ginger straw, the main byproduct in the ginger planting process, boasts a huge yield. In this study, ginger straw substrate (GSS) was assessed for the first time for cultivating five major edible fungi. The results indicated a significant improvement in biological efficiency (BE) with GSS, increased by 1.22–64.81%. In terms of nutritional properties, the GSS not only significantly increased the crude protein content (0.36~10.6%) and reduced sugar content (0.01~1%), crude fiber content (0.14~3.87%), and mineral level (The maximum increases were 217.02 mg/kg for calcium, 4.74 mg/kg for magnesium, and 44.08 mg/kg for iron) but also positively affected the total antioxidant capacity and composition of flavor-contributing amino acids. These results provide a scientific basis for cultivating edible fungi with ginger straw and offer a new way for edible fungi substrate selection.

## 1 Introduction

Edible fungi are hailed as the “Food of the Gods” (1, 2). They possess a significant nutritional value, attributed to their abundance of protein and a notable presence of essential amino acids, dietary fiber, and vitamins (3–5). Hence, edible fungi emerge as a superior source of diverse nutraceuticals and might be used directly in the human diet to enhance health (6–9). Modern medical research uncovered a plethora of over 100 bioactive compounds in edible fungi, including polysaccharides, terpenoids, alkaloids, flavonoids, lectins, and organic acids. These compounds contribute to a multitude of health benefits, for instance, antioxidant, anticancer, anti-allergic, immunomodulatory, cardioprotective, anticholesterolemic, and hepatoprotective activities (10). More significantly, edible fungi employ agricultural wastes, such as cottonseed hulls and wheat bran, as their cultivation substrate and convert these waste materials into healthy and delicious food. Furthermore, the spent edible fungi substrate can be further processed into fertilizer, animal feed, or biogas production (11–14). Therefore, edible fungi, as a kind of agricultural food resource with unique nutritional and economic value, is gradually showing great potential in modern agriculture and food industry.

Since 1990, after 30 years of development, by 2020, the global edible fungi industry has achieved remarkable progress. The output of edible fungi has increased by 13.8 times compared with that in 1990, and finally, the output of edible fungi reached as high as 42.8 million tons in 2020, which led to a sharp increase in the demand for cultivation substrate materials (15). At present, the types of substrate materials commonly used in edible fungi cultivation are relatively limited, including sawdust, cottonseed hulls, corn cobs, and wheat bran. These cultivated materials mainly provide carbon sources (cellulose, hemicellulosic, lignin, etc.) and nitrogen sources for the growth of edible fungi. However, the supply of these materials is influenced by various factors, such as geography, climate, and seasons. Additionally, some materials, such as sawdust, are limited by forestry resources. Therefore, there is an urgent need to develop new substrates for edible fungi (16). Ginger is widely cultivated as a significant economic crop. The United Nations’ Food and Agriculture Organization (FAO) reported that ginger was cultivated by over 50 countries, and as of 2023, ginger production over the world is approximately 48.9 billion tons. China was one of the core areas of the world’s ginger industry, and the planting area was stable at approximately 270,000 hectares. A large amount of straw is produced during the harvest of ginger, whose integrated management and safe disposal is particularly challenging due to the huge volume, seasonality in production, and high organic load. It has been reported that ginger straw contains rich nutrients, including protein, cellulose, hemicellulose, lignin, and various trace elements (17), making it a potential substrate for edible fungi cultivation. Previous research has shown that different cultivation substrates can lead to variations in the nutrition and flavor of edible fungi. However, there have been no reports on the utilization of ginger straw as a substrate for the cultivation of edible fungi (18–23), and no data exist on the effect of ginger straw substrate (GSS) on the yield, fruiting body traits, and nutritional properties of edible fungi. Hence, the main objectives of this study were (a) to investigate the suitability of GSS as novel substrates for five leading species of edible fungi. These species were *Pleurotus ostreatus*, *Flammulina filiformis*, *Auricularia heimuer*, *Auricularia cornea*, and *Pleurotus eryngii*, each producing over 1 million tons annually in China (24); and (b) to assess the impact of the substrate on the yield, fruiting body traits, and nutritional properties of five edible fungi to determine their production and exploitation potential.

## 2 Materials and methods

### 2.1 Edible fungi material and spawn preparation

Five representative edible fungi were focused on as experimental subjects in this study. Extensive collection and screening of the primary cultivated strains of these edible fungi were undertaken nationwide. Finally, totaling 17 strains were selected for this study, including P1, P3, P4, P6, and P7 of *P. ostreatus*, J1, J5, and J7 of *F. filiformis*, XB2, XB3, XB4, and XB5 of *P. eryngii*, h2, h4, and h7 of *A. heimuer*, and M2 and M6 of *A. cornea* (Table 1; Supplementary material) (25).

**Table 1:** The strains used in this study.

Edible fungi	Strain name in this study	The original name of strains	Sources
*P. ostreatus*	P1	CCMSSC 00374	Institute of Agricultural Resources and Regional Planning, CAAS
	P3	CCMSSC 04195	Institute of Agricultural Resources and Regional Planning, CAAS
	P4	CCMSSC 0406	Institute of Agricultural Resources and Regional Planning, CAAS
	P6	*P. ostreatus*-1	Jilin Agricultural University
	P7	*P. ostreatus*-2	Jilin Agricultural University
*F. filiformis*	J1	CCMSSC 00104	Institute of Agricultural Resources and Regional Planning, CAAS
	J5	Vary 19	Department of Mycology, College of Plant Protection, Shandong Agricultural University
	J7	2,793	Jilin Agricultural University
*P. eryngii*	XB2	1,067	Jilin Agricultural University
	XB3	1,071	Jilin Agricultural University
	XB4	1,101	Jilin Agricultural University
	XB5	1,104	Jilin Agricultural University
*A. heimuer*	h2	H02	Jilin Agricultural University
	h4	H-1C	Jilin Agricultural University
	h7	F7H08	Jilin Agricultural University
*A. cornea*	M2	CCMSSC04337	Institute of Agricultural Resources and Regional Planning, CAAS
	M6	*A. cornea* 2020	Jilin Agricultural University

Strains of edible fungi were cultivated using a PDA (Potato Dextrose Agar) substrate in this study. The substrate was formulated with 200.0 g of peeled potatoes, 20.0 g of glucose, 20.0 g of agar, 3.0 g of KH_2_PO_4_, and 1.5 g of MgSO_4_•7H_2_O, and the volume was adjusted to 1 L with deionized water. The substrate was then subjected to autoclaving at 121°C for 30 min. Under sterile conditions on a clean bench, the substrate was poured into sterile petri dishes. Holes were punched on the substrate’s surface using an alcohol-flamed puncher, and into these, pre-cultured fungal plugs were inoculated using a similarly flamed and cooled inoculation needle.

### 2.2 Substrate preparation and fruiting test

Optimal addition ratios for each strain were revealed through a comprehensive analysis of growth rate and vigor in culture substrate containing increments of 10% to 40% GSS (Supporting Information). Fruiting trials based on the screened ratios were conducted using the formulations specified (Table 2). Ginger straw was dried and broken into 1 cm particles by fragmentation. The GSS was prepared and loaded into 10 × 23 cm polyethylene bags (Table 2). Subsequently, autoclaving was conducted at 121°C and 0.12~0.14 MPa for 3 h, followed by cooling to room temperature. Ten bags containing either GSS or conventional cottonseed hull culture substrate were inoculated with each strain and subsequently placed in a mycelium cultivation room. Conditions were maintained at 28°C with a relative humidity of 60~70% in the dark room. Once the mycelium had fully colonized the bags, they were transferred to a planting room where the temperature was maintained at 24°C and the relative humidity was kept above 90%.

**Table 2:** Cultivation substrate formula (mass fraction).

Edible fungi	Group	Formulations
*P. ostreatus*	P1 (0)	Cottonseed hulls 90%, wheat bran 8%, quicklime 2%
	P1 (25)	Ginger straw 25%, cottonseed hulls 65%, wheat bran 8%, quicklime 2%
	P3 (0)	Cottonseed hulls 90%, wheat bran 8%, quicklime 2%
	P3 (35)	Ginger straw 35%, cottonseed hulls 55%, wheat bran 8%, quicklime 2%
	P4(0)	Cottonseed hulls 90%, wheat bran 8%, quicklime 2%
	P4(35)	Ginger straw 35%, cottonseed hulls 55%, wheat bran 8%, quicklime 2%
	P6(0)	Cottonseed hulls 90%, wheat bran 8%, quicklime 2%
	P6(35)	Ginger straw 35%, cottonseed hulls 55%, wheat bran 8%, quicklime 2%
	P7(0)	Cottonseed hulls 90%, wheat bran 8%, quicklime 2%
	P7(30)	Ginger straw 30%, cottonseed hulls 60%, wheat bran 8%, quicklime 2%
*F. filiformis*	J1(0)	Cottonseed hulls 87%, wheat bran 11%, quicklime 2%
	J1(25)	Ginger straw 25%, cottonseed hulls 62%, wheat bran 11%, quicklime 2%
	J5(0)	Cottonseed hulls 87%, wheat bran 11%, quicklime 2%
	J5(20)	Ginger straw 20%, cottonseed hulls 67%, wheat bran 11%, quicklime 2%
	J7(0)	Cottonseed hulls 87%, wheat bran 11%, quicklime 2%
	J7(20)	Ginger straw 20%, cottonseed hulls 67%, wheat bran 11%, quicklime 2%
*P. eryngii*	XB2(0)	Cottonseed hulls 93%, wheat bran 6%, quicklime 1%
	XB2(20)	Ginger straw 20%, cottonseed hulls 73%, wheat bran 6%, quicklime 1%
	XB3(0)	Cottonseed hulls 93%, wheat bran 6%, quicklime 1%
	XB3(25)	Ginger straw 25%, cottonseed hulls 68%, wheat bran 6%, quicklime 1%
	XB4(0)	Cottonseed hulls 93%, wheat bran 6%, quicklime 1%
	XB4(30)	Ginger straw 30%, cottonseed hulls 63%, wheat bran 6%, quicklime 1%
	XB5(0)	Cottonseed hulls 93%, wheat bran 6%, quicklime 1%
	XB5(35)	Ginger straw 35%, cottonseed hulls 58%, wheat bran 6%, quicklime 1%
*A. heimuer*	h2(0)	Cottonseed hulls 78%, wheat bran 20%, gypsum 1%, sucrose 1%
	h2(20)	Ginger straw 20%, cottonseed hulls 58%, wheat bran 20%, gypsum 1%, sucrose 1%
	h4(0)	Cottonseed hulls 78%, wheat bran 20%, gypsum 1%, sucrose 1%
	h4(20)	Ginger straw 20%, cottonseed hulls 58%, wheat bran 20%, gypsum 1%, sucrose 1%
	h7(0)	Cottonseed hulls 78%, wheat bran 20%, gypsum 1%, sucrose 1%
	h7(20)	Ginger straw 20%, cottonseed hulls 58%, wheat bran 20%, gypsum 1%, sucrose 1%
*A. cornea*	M2(0)	Cottonseed hulls 75%, wheat bran 20%, quicklime 3%, calcium superphosphate 1%, light calcium powder 1%
	M2(15)	Ginger straw 15%, cottonseed hulls 63%, wheat bran 20%, quicklime 3%, calcium superphosphate 1%, light calcium powder 1%
	M6(0)	Cottonseed hulls 75%, wheat bran 20%, quicklime 3%, calcium superphosphate 1%, light calcium powder 1%
	M6(35)	Ginger straw 35%, cottonseed hulls 43%, wheat bran 20%, quicklime 3%, calcium superphosphate 1%, light calcium powder 1%

Group represents: strain name (ginger straw addition ratio, %).

Fruiting bodies were harvested. The time from inoculation to the first harvest and total harvesting time (from the first to the last harvest) were observed and recorded.

### 2.3 Determination of fruiting body traits

The harvested fruiting bodies were weighed using an electronic scale to calculate the total yield and BE. BE is the ratio of fresh fruiting body weight (g) per dry weight of substrates (g), expressed as a percentage.

Vernier calipers were used to measure the cap thickness, cap diameter, and stipe length of fresh samples from fruiting bodies. The moisture content of the fruiting bodies was measured by employing the direct drying method, as stipulated by national standard GB 5009.3–2016. Fruiting bodies are dried in an oven at a predetermined temperature until a constant weight is achieved, and the moisture content is then calculated based on the mass difference before and after drying.

### 2.4 Nutritional component analysis in fruiting bodies

During sample processing, fresh samples are exposed to a well-ventilated environment for natural air-drying over 2 days. Subsequently, the samples are dried in an oven set at 50°C until a constant weight is achieved. The cut, dried fruiting bodies were ground into a powder, and the powdered fruiting bodies were stored at 4°C until being analyzed.

#### 2.4.1 Crude protein content

Crude protein content is measured in accordance with GB 5009.5–2016, “Determination of protein in foods by kjeldahl method,” using a conversion factor of 4.38.

#### 2.4.2 Crude fat content

Crude fat content determination follows GB 5009.6–2016, “Determination of fat in foods by soxhlet extraction method.”

#### 2.4.3 Total sugar content

The determination of total sugar content in edible fungi is carried out in accordance with the national standard GB/T15672-2009, “Determination of total sugar content in edible fungi.”

#### 2.4.4 Reducing sugar content

Reducing sugar content was determined according to the direct titration by the China national food standard GB 5009.7–2016.

#### 2.4.5 Ash content

Ash content was determined according to the high-temperature burning method by the China national food standard GB 5009.4–2016.

#### 2.4.6 Crude fiber content

Crude fiber content was determined according to the method of acid-base treatment by the China national food standard GB 5009.10–2003.

#### 2.4.7 Antioxidant activity assays

The antioxidant activities of five edible fungi were examined for their response to varying ginger straw addition ratios in the cultivation substrate using the ferric reducing antioxidant power (FRAP) assay.

#### 2.4.8 Determination of amino acid composition

Amino acid analysis of the edible fungi was performed according to the Chinese national standard, GB 5009.124–2016. In brief, 100 mg of dried fruiting bodies was hydrolyzed in screw-capped glass test tubes for 22 h at 110 ± 1°C using 10 mL of 6 mol/L HCl. The acid hydrolyzate was filtered through filter paper and evaporated using a tube concentrator at 50°C under reduced pressure. Subsequently, 1.0 mL sodium citrate buffer solution (pH 2.2) was added to the tube to re-dissolve the dried hydrolyzate. The obtained solution was passed through a 0.22 mL filter membrane and used to determine amino acids. The sample solution and amino acid standard working solution were separately injected into the amino acid analyzer, and the content of amino acids in the sample solution was calculated through the peak area using the external standard method.

#### 2.4.9 Trace element content

The content of minerals and trace elements in the fruiting bodies was determined by an inductively coupled plasma emission spectrometer, in accordance with the national food safety standard GB 5009.268–2016 “Determination of multielements in foods.” Minerals and trace elements, including Ca, Cu, Mn, Fe, Zn, Cd, and Pb, were subjected to analysis. Detection limits for each mineral and trace element were established, and quantitative analyses were conducted under suitable instrumental conditions. The content of minerals and trace elements in powdered fruiting bodies are recorded in mg/kg.

### 2.5 Evaluation of the nutritional value of fruiting body proteins

①Amino acid scoring (AAS), AAS = w1/w2 × 100. Formula: w1 is the content of a specific amino acid in the protein being evaluated, in mg/g; w2 is the content of the corresponding amino acid in the reference protein pattern, in mg/g.

②Chemical score (CS), CS = w1/w2 × w3/w4 × 100. Formula: w1 is the content of a specific EAA in the protein being evaluated, in mg/g; w2 is the total content of EAA in the protein being evaluated, in mg/g; w3 is the content of the corresponding EAA in the reference protein, in mg/g; w4 is the total content of EAA in the reference protein, in mg/g.

③Essential Amino Acid Index (EAAI), EAAI = (A/AE × B/BE × … × I/IE)^(1/n) Formula: n is the number of EAA tested; A, B, …, I are the contents of EAA in the protein under test; AE, BE, …, IE are the contents of EAA in egg protein, in mg/g.

### 2.6 Comprehensive evaluation of fruiting bodies using membership function method

The membership function method is employed to conduct a comprehensive and systematic evaluation of the comprehensive quality of specific strains in fruiting bodies. The calculation formula is as follows:


U(Xi)=(Xi−Xmin)/(Xmax−Xmin)

In the equation, the value of the membership function method is denoted by U(Xi). An indicator specified by the index i has Xi as its measured value. The maximum and minimum values of this indicator are represented by Xmax and Xmin, respectively. Once each indicator for the sub-entity has been calculated, the values are aggregated and averaged to derive the comprehensive quality measurement of the strain. Comprehensive quality measurements for the sub-entities of ginger straw and cottonseed hull are calculated independently. The influence of the ginger straw culture substrate on the sub-entity’s comprehensive quality is assessed by comparing these measurements.

### 2.7 Statistical analysis

Data were expressed as mean ± standard deviation (SD). IBM SPSS Statistics for Windows, version 27.0 (IBM Corp, Armonk, NY, United States), was used for the statistical analyses (26). One-way analysis of variance (ANOVA) was used for multiple comparisons. Statistical significance is represented by different letters corresponding to *p* < 0.05 based on Duncan’s test. TBtools-II (27), R Core (version 4.2.3; R Core Team, 2022) (28), and GraphPad Prism version 10.1.2 for Windows (GraphPad Software, San Diego, CA, United States)^1^ were used for drawing graphics.

## 3 Results and discussion

In our study, we selected the most commonly used strains in production. The five of the most common commercial edible fungi and their common production strains (with an annual output of more than 1 million tons in China) were selected for the experiment, including *P*. *ostreatus*, *F. filiformis*, *P. eryngii*, *A. heimuer*, and *A. cornea* (29). The main cultivated strains of these five edible fungi in China were extensively collected and screened, and 17 strains were ultimately selected for this experiment. By comprehensively analyzing the mycelium growth conditions and ginger straw utilization rate in media with ginger straw addition ratios 10, 15, 20, 25, 30, 35, and 40%, an optimal ginger straw addition proportion was selected for each strain to conduct fruiting experiments. The process and results are shown in the Supplementary material. The optimal ginger straw addition ratios for strains P1, P3, P4, P6, and P7 of *P. ostreatus* were determined to be 25, 35, 35, 35, and 30%, respectively. The strains J1, J5, and J7 of *F. filiformis* had optimal ginger straw addition ratios of 25%, 25%, and 20%, respectively. Optimal ginger straw addition ratios for strains XB2, XB3, XB4, and XB5 of *P. eryngii* were 20, 25, 30, and 35%. The proportion of ginger straw addition in h2, h4, and h7 strains of *A. heimuer* was 20%. Strains M2 and M6 of *A. cornea* had optimal ginger straw addition ratios of 15 and 35%, respectively. Culture substrates were prepared according to the above ginger straw addition ratios, and fruiting experiments were conducted on five edible fungi.

### 3.1 The effect of GSS on the biological efficiency and fruiting body traits of five edible fungi

#### 3.1.1 The effect of GSS on the biological efficiency

The biological efficiency (BE) of five edible fungi grown in ginger straw culture substrate was analyzed, with conventional cottonseed hull culture substrate serving as a control. The results indicated that the BE of the five edible fungi was significantly influenced by the GSS, generally demonstrating an increasing trend (Table 3). However, variations were observed in the impact on BE among different strains of the same species. BE of *P. ostreatus* ranges from 70.74 to 147.59%. Compared to the control, the BE of strains P4, P6, and P7 exhibited increases of 19.48, 60.61, and 64.81%, respectively, whereas strain P3 showed a decrease of 1.13%. A BE ranging from 105.85 to 109.22% was exhibited by *F. filiformis*. Increases of 4.07, 4.28, and 2.88% in the BE of strains J1, J5, and J7 were observed compared to the control, though these were not significant. The BE ranging from 61.69 to 76.38% was observed in *P. eryngii*. For strain XB4, an increase of 27.46% over the control was recorded, in contrast to strains XB2, XB3, and XB5, where no significant changes were noted. The BE of *A. heimuer* varied from 73.64 to 74.55%, with strain h7 exhibiting a significant increase of 2.15%, whereas strains h2 and h4 saw no significant changes. Strains M2 and M6 of *A. corneas* had BE ranging from 74.03 to 66.82%, showing no significant improvements compared to the control. The cultivation of edible fungi using agricultural waste is a prominent research topic in current sustainable agriculture. It was discovered by Muswati et al. that the highest BE, 86.15%, was yielded by cotton waste combined with wheat straw for cultivating *P. ostreatus*, while the lowest efficiency, 42.5%, resulted from baobab fruit shells mixed with wheat straw (30). BE ranging from 71.95 to 88.66% was observed in *P. ostreatus* cultivated with varying ratios of rice straw and sugarcane bagasse in experiments conducted by De et al. (31). A maximum BE of 79.7% was recorded in Oliveira do Carmo’s et al. study, where *P. ostreatus* were cultivated with varying ratios of chopped sisal leaves and dry fiber powder waste (32). The results of these experiments were surpassed by the BE of *P. ostreatus* fruiting bodies cultivated with ginger straw in this study. Additionally, experiments conducted by Zhou et al. with varying proportions of sawdust, sugarcane bagasse, and corn straw in substrates for cultivating *P. eryngii* resulted in BE ranging from 68.4 to 78.71%. BE observed in this research were found to be comparable to those of *P. eryngii* cultivated with ginger straw (18). A BE of under 10% was recorded in experiments by Hao et al. using various ratios of mixed and walnut wood chips for *A. heimuer* cultivation, significantly lower than that achieved with ginger straw (33). The BE of edible fungi is influenced by the type and amount of agricultural waste additives used. The variations are likely attributed to differences in the physical and chemical compositions, including the cellulose, lignin, and mineral content and proportions, of the various agricultural wastes. Overall, GSS positively influences the BE of edible fungi, such as *P. ostreatus* and *P. eryngii*, compared to other agricultural wastes.

**Table 3:** Biological efficiency and fruiting body traits of five edible fungi on the cottonseed hull substrate and GSS.

Group	BE (%)	Length of stipe (mm)	Diameter of cap (mm)	Thickness of cap (mm)	Water content (%)
P1 (0)	108.64 ± 13.3 c	49.85 ± 1.16 def	47.03 ± 2.69 de	2.06 ± 0.32 c	92.46 ± 0.62 a
P1 (25)	104.62 ± 0.41 c	49.89 ± 2.43 def	46.23 ± 1.12 de	2.44 ± 0.06 c	90.48 ± 0.49bc
P3 (0)	71.87 ± 9.31 e	42.01 ± 3.06 g	35.29 ± 2.34 g	2.37 ± 0.18 c	90.54 ± 0.31bc
P3 (35)	70.74 ± 4.11 f	40.89 ± 4.04 gh	51.46 ± 1.62 cd	2.27 ± 0.05 c	90.78 ± 0.39bc
P4(0)	88.85 ± 1.67 d	52.98 ± 14.35 de	54.69 ± 7.06 c	2.95 ± 1.29bc	91.03 ± 0.29 b
P4(35)	108.33 ± 6.81 c	52.74 ± 3.99 de	51.43 ± 1.29 cd	3.15 ± 0.23 c	90.48 ± 0.02bc
P6(0)	73.31 ± 2.77 de	54.22 ± 1.94 d	59.78 ± 1.56 b	3.37 ± 0.25 b	89.9 ± 0.49 c
P6(35)	133.92 ± 3.38 b	60.49 ± 3.9 cd	39.86 ± 0.57 fg	4.28 ± 0.17 c	90.52 ± 0.4 bc
P7(0)	82.78 ± 5.96 de	66.19 ± 2.5 bcd	80.11 ± 0.44 a	3.31 ± 0.02 a	90.86 ± 1.22bc
P7(30)	147.59 ± 1.82 a	69.1 ± 3.72 a	43.21 ± 0.83 ef	4.65 ± 0.39bc	90.11 ± 0.1 bc
J1(0)	101.78 ± 1.36 c	126.56 ± 9.65 a	5.54 ± 0.25 a	3.6 ± 0.18 bc	89.63 ± 0.03 a
J1(25)	105.85 ± 0.48abc	153.38 ± 3.56 a	4.94 ± 0.07 bc	4.27 ± 0.07 a	88.55 ± 0.14 c
J5(0)	103.91 ± 0.92 bc	147.23 ± 6.33 a	4.34 ± 0.21 d	3.52 ± 0.05 c	89.61 ± 0.02 a
J5(20)	108.19 ± 0.46 ab	156.83 ± 5.39 a	4.58 ± 0.33 cd	3.77 ± 0.35bc	89.29 ± 0.14 b
J7(0)	106.34 ± 3.6 abc	142.36 ± 20.54a	5.15 ± 0.16 ab	4 ± 0.09 ab	88.61 ± 0.12 c
J7(20)	109.22 ± 2.99 a	147.83 ± 6.67 a	4.71 ± 0.23bcd	3.87 ± 0.05bc	89.65 ± 0.09 a
XB2(0)	56 ± 1.73 bc	87.42 ± 3.96 ab	51.16 ± 0.27 a	11.33 ± 0.18c	88.29 ± 0.8 ab
XB2(20)	64.08 ± 3.64 bc	95.99 ± 11.58 a	57.70 ± 2.12 a	12.51 ± 0.68c	90.07 ± 0.19 a
XB3(0)	46.15 ± 5.33 d	73.92 ± 6.7 b	53.03 ± 0.33 a	11.22 ± 0.05c	89.71 ± 1.74 a
XB3(25)	61.69 ± 6.62 cd	77.25 ± 3.21 b	52.6 ± 4.92 a	14.94 ± 1.26b	89.86 ± 0.09ab
XB4(0)	48.92 = ±0.11 cd	76.53 ± 5.47 b	53.86 ± 0.48 a	11.86 ± 0.16c	88.52 ± 0.53ab
XB4(30)	76.38 = ±2.71 a	86.39 ± 2.26 ab	55.61 ± 6.44 a	12.54 ± 1.18c	88.59 ± 0.11ab
XB5(0)	67.31 ± 4.85 ab	85.19 ± 3.32 ab	55.57 ± 0.79 a	12.32 ± 0.66c	86.86 ± 1.75 b
XB5(35)	69.31 ± 10.05 bc	95.74 ± 4.3 a	59.55 ± 4.06 a	14.81 ± 0.33a	90.16 ± 1.48 a
h2(0)	73.66 ± 1.04 ab	–	45.72 ± 1.62 a	4.1 ± 0.21 a	90.38 ± 0.54ab
h2(20)	73.64 ± 0.53 ab	–	44.83 ± 4.58 a	4.21 ± 0.07 a	90.84 ± 0.29 a
h4(0)	73.33 ± 0.57 ab	–	45.64 ± 3.89 a	4.15 ± 0.1 a	89.2 ± 0.54 bc
h4(20)	74.55 ± 0.1 a	–	43.93 ± 1.28 b	4.18 ± 0.1 a	88.7 ± 0.11 c
h7(0)	72.26 ± 0.51 b	–	45.23 ± 0.69 a	4.2 ± 0.05a	90.52 ± 0.11ab
h7(20)	74.41 ± 1.39 a	–	44.17 ± 3.78 b	4.17 ± 0.11a	90.68 ± 0.29 a
M2(0)	74.23 ± 2.89 a	–	44.45 ± 0.29 a	4.1 ± 0.21 a	86.89 ± 0.5 ab
M2(15)	74.03 ± 1.41 a	–	42.34 ± 0.45 b	4.21 ± 0.07 a	85.46 ± 0.1 b
M6(0)	70.21 ± 2.87 ab	–	42.79 ± 0.33 b	4.08 ± 0.1 a	87.54 ± 0.63 a
M6(35)	66.82 ± 2.01 b	–	44.09 ± 0.54 a	4.18 ± 0.1 a	85.47 ± 0.91 b

“–”: The traits are not applicable to this strain. Group represents: strain name (ginger straw addition ratio, %). Different lowercase letters indicate significant differences, *p* < 0.05.

#### 3.1.2 The effect of GSS on fruiting body traits

The fruiting body traits of five edible fungi were measured (Table 3). The stipe lengths of *P. ostreatus*, *F. filiformis*, and *P. eryngii* generally increased compared to the control group. The stipe lengths of *P. ostreatus* ranged from 40.89 to 69.10 mm. The stipe lengths in the P6 and P7 treatment groups significantly increased by 6.27 and 2.91 mm, respectively, compared to the control group. *F. filiformis* had stipe lengths ranging from 147.83 to 156.83 mm, while *P. eryngii* ranged from 77.25 to 95.99 mm. Although the stipe lengths of these two edible fungi increased, the growth did not reach a statistically significant level. In the study by Fufa et al., *P. ostreatus* grown on substrates containing varying ratios of sawdust, sugarcane bagasse, and corn bran exhibited stipe lengths between 35.28 and 38.34 mm, shorter than those grown on the GSS (34). On the other hand, the cap diameters of *P. ostreatus*, *F. filiformis*, and *P. eryngii* were 39.86–51.46 mm, 4.58–4.94 mm, and 52.6–59.55 mm, respectively, showing no clear pattern in their changes. The cap diameters of *A. heimuer* and *A. cornea* generally decreased. Specifically, the cap diameters of *A. heimuer* ranged from 43.93 to 44.83 mm, with significant reductions of 1.71 and 1.06 mm for strains h4 and h7 compared to the control; the cap diameters of *A. corneas* were 42.34 and 44.09 mm, with a significant reduction of 2.11 mm for strain M2. The thicknesses of the caps for *P. ostreatus*, *F. filiformis*, *P. eryngii*, *A. heimuer*, and *A. cornea* were 2.27–4.65 mm, 3.52–3.87 mm, 12.51–14.94 mm, 4.17–4.21 mm, and 4.21 mm and 4.18 mm, respectively, all showing a general trend of increase. Specifically, the cap thicknesses of *P. ostreatus* in treatment groups P1, P4, P6, and P7 increased significantly, being 0.38, 0.2, 0.91, and 1.34 mm thicker than the control group, respectively; the cap thickness of *F. filiformis* in treatment group J1 also increased significantly by 0.67 mm; the cap thicknesses of *P. eryngii* in treatment groups XB3 and XB5 increased significantly by 3.72 and 2.49 mm, respectively; except for a slight decrease in the cap thickness of *A. heimuer* in treatment group h7, the increases in cap thickness in the other treatment groups of *A. heimuer* and all treatment groups of *A. cornea* were not significant. Additionally, the ginger straw culture substrate had a certain impact on the water content of five edible fungi, which ranged from 85.46 to 90.86%. However, no clear regularity was observed. These results were similar to those obtained by Bonatti et al., who used rice straw and banana straw for cultivating *P. ostreatus*, with water content of 88.06 and 85.64%, respectively (35). These experimental findings further demonstrate the suitability and potential of ginger straw as a cultivation substrate.

### 3.2 The effect of GSS on the nutritional quality of fruiting bodies of five edible fungi

#### 3.2.1 The effect of GSS on crude fat, crude protein, total sugar, reducing sugar, crude fiber, and ash content

**Figure 1:**
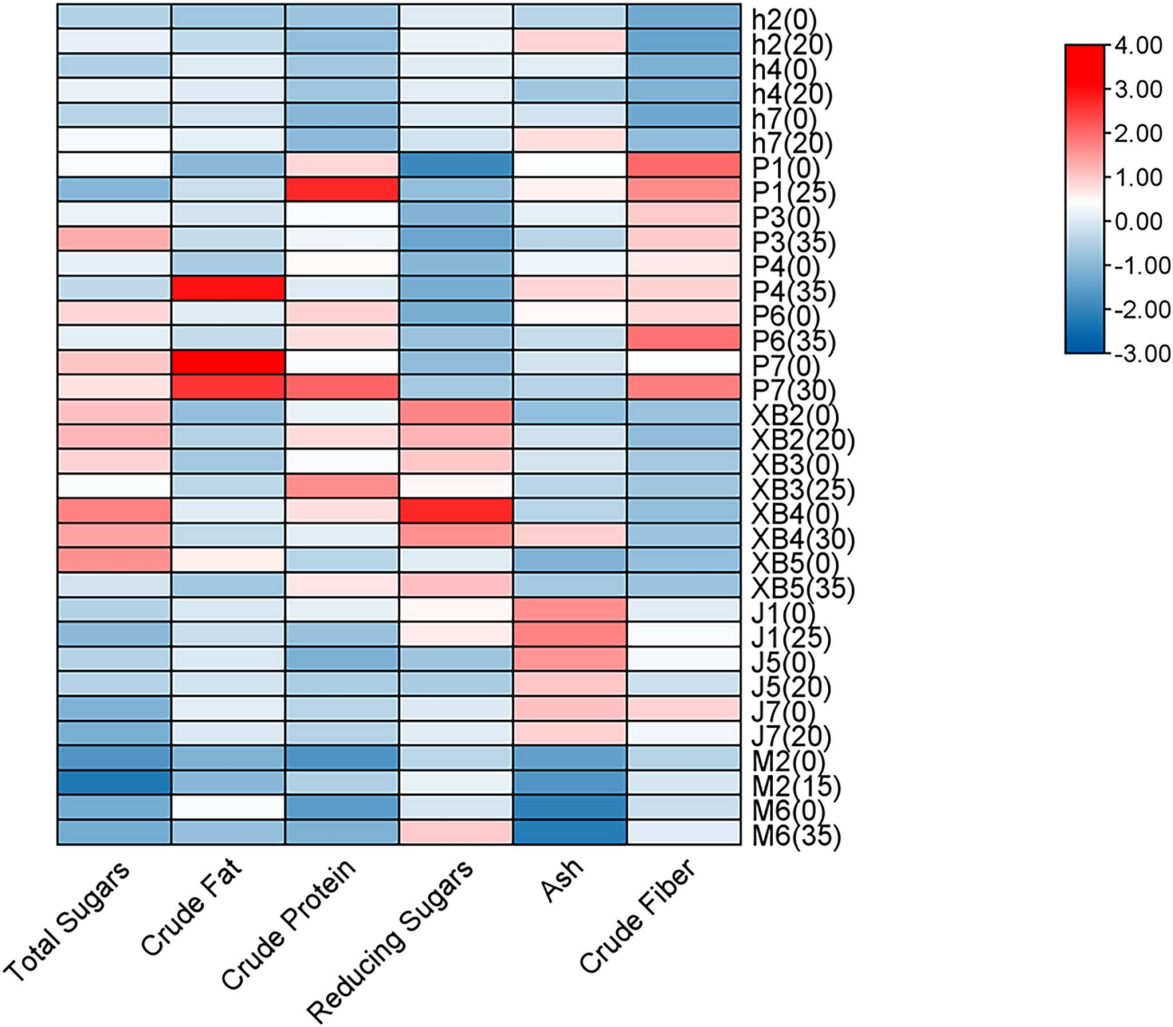
Heat map analysis of the nutritional components of five edible fungi on the cottonseed hull substrate and GSS.

Crude fat, protein, total and reducing sugars, fiber, and ash are key nutritional indicators in edible fungi. In this study, the effects of GSS on these nutritional components in five edible fungi were investigated, with the fruiting bodies cultivated on conventional cottonseed hull substrate serving as the control (Figure 1).

Overall, crude protein levels were significantly increased by the ginger straw culture substrate. In *P. ostreatus*, crude protein levels ranging from 16.80 to 31.98% were observed, with increases of 10.59% in strain P1 and 8.83% in strain P7 being noted. Crude protein levels ranging from 17.08 to 25.94% were observed in *P. eryngii*, with gains of 7.28% in XB3 and 6.43% in XB5 being significant. Crude protein levels of 13.62 and 10.11% were recorded in *A. cornea*, with improvements of 6.93 and 2.39% over the control group being significant. It was reported by Koutrotsios et al. that crude protein levels in *P. ostreatus* grown on substrates of nut shells, beech sawdust, corn cobs, and olive press cake ranged only from 0.3 to 14.2%. Although comparable to levels from substrates mixed with grape pomace and cotton gin trash at 17.1%, these were significantly lower than those achieved with ginger straw in this study (36).

The increase in crude fiber content was also notable. The crude fiber content of *P. ostreatus* ranged from 7.76 to 12.36%, with highly significant increases of 3.13 and 3.87% observed in strains P6 and P7, respectively. In the ginger straw culture substrate, the crude fiber content of *A. cornea* was 6.54 and 6.87%, with the fruiting bodies of strains M2 and M6 showing highly significant increases of 1.1 and 0.83%, respectively. Although these increases were slightly lower than the range of crude fiber content achieved in Koutrotsios’ et al. study using a mixed substrate of nut shells, beech sawdust, and corn cobs (23.2–50.9%), they were close to the crude fiber content of *P. ostreatus* cultivated on pine needle substrates (11.6%) (36).

Additionally, an enhancement in reducing sugar content was observed in the ginger straw culture substrate compared to the conventional cottonseed hull culture substrate. Reducing sugar content ranging from 1.63 to 2.29% was observed in *P. ostreatus*, with highly significant increases of 1.15, 0.39, and 0.31% recorded in strains P1, P6, and P7, respectively. In *A. cornea*, reducing sugar levels of 3.05 and 3.82% were recorded, with highly significant increases of 0.99 and 0.51% observed in strains M6 and M2, respectively. A significant surpassing of reducing sugar levels between 0.62 and 0.85%, as recorded in *A. heimuer* grown on sawdust and corn straw substrates from the earlier study by Yao et al., was noted, underscoring the substantial benefit of ginger straw in enhancing sugar accumulation in edible fungi (37). However, the total sugar content, which varied from 10.13 to 42.79% in five edible fungi, was generally found to decrease with the use of ginger straw culture substrate. Significant reductions, ranging from 2.7 to 15.26%, were recorded in the sugar content of several *P. ostreatus* varieties (P1, P4, P6, and P7) and *P. eryngii* (XB3 and XB5). The decrease may be attributed to the rapid release of sugars by the ginger straw, which is believed to facilitate a quicker conversion of sugars into biomass or other metabolites by the edible fungus. Conversely, a significant increase in the sugar content of *A. heimuer* was observed, which could be attributed to variations in metabolic pathways and substrate utilization among different edible fungi.

Ash content was significantly reduced across various edible fungi by the ginger straw culture substrate. In *P. ostreatus*, ash content was found to range from 6.22 to 8.21%, with reductions of 0.84 and 1.16% observed in the P3 and P6, respectively. In *F. filiformis*, ash content was recorded from 8.28 to 8.55%, with a 0.6% decrease noted in the J5 strain and 0.3% in both the J1 and J7 strains. Ash content in *A. heimuer* was observed to range between 5.84 and 8.24%, with a significant reduction of 1.1% being noted in the h4. These ash levels were comparable to those identified in *P. ostreatus* grown on rice and banana straw (5.14–6.13%) by Bonatti (35) and on the substrate of soybean, rice, sunflower, and peanut straws (5.9–7.0%) as reported by Patil (38). The viability and effectiveness of ginger straw as a substrate for cultivating edible fungi were supported by this evidence.

Finally, crude fat content was observed to vary between 1.94 and 10.34% in five edible fungi. No discernible pattern was demonstrated in the influence of the ginger straw culture substrate on the crude fat content of these edible fungi. Increases and significant decreases in crude fat content were observed in P1 and P4 of *P. ostreatus*, h7 and h2 of *A. heimuer*, XB5 of *P. eryngii*, and M6 of *A. cornea*, respectively. This inconsistency may be attributed to various factors. Despite irregular changes, the crude fat content in the *P. ostreatus* (3.3% to 10.3%) was found to be comparable to or even higher than levels reported by Patil, who utilized rice, wheat, peanut, and sunflower straw as culture substrates (38). The crude fat content in *P. eryngii* (2.60–3.43%) was also found to closely match that in edible fungus grown using rice straw and corn cob substrates by Sardar et al. (2.6–3.4%) (39).

In summary, ginger straw demonstrates significant advantages and potential as a substrate for cultivating edible fungi. This substrate not only enhances the content of reducing sugars in edible fungus but also uniquely manages total sugars and ash content. Despite irregular changes in crude fat content, ginger straw offers a new and sustainable substrate for producing high-quality edible fungi.

#### 3.2.2 The effect of GSS on the total antioxidant capacity

The antioxidant properties of edible fungi are derived from high levels of flavonoids, polyphenols, and other antioxidants, which are known to effectively scavenge free radicals, inhibit lipid peroxidation, activate antioxidant enzymes, and chelate harmful metal ions. Cells are shielded from oxidative damage, aging is slowed, and diseases are prevented by these compounds in edible fungi.

**Figure 2:**
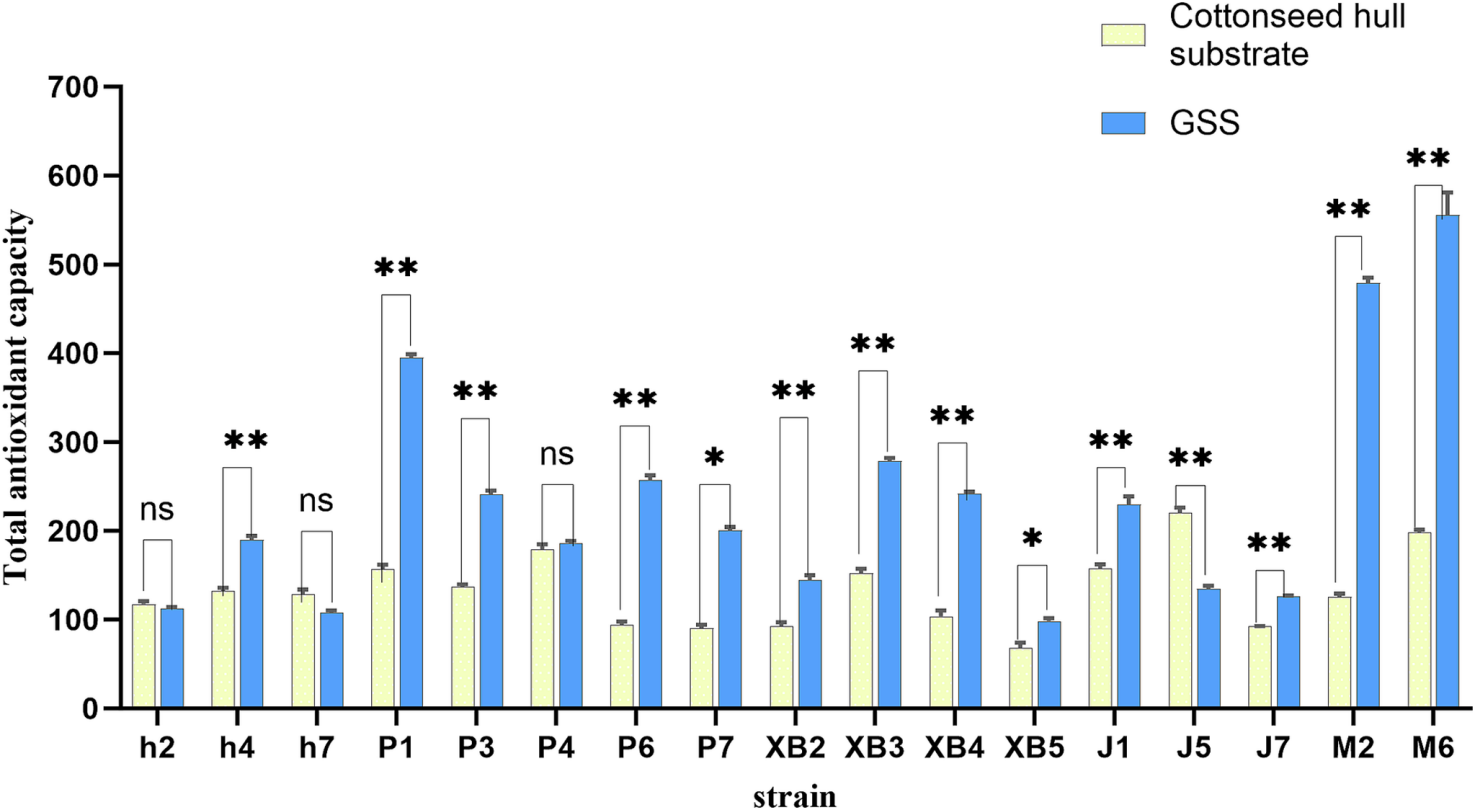
Total antioxidant capacity of five edible fungi on the cottonseed hull substrate and GSS. *p* < 0.05. ns represents insignificant difference, * represents significant difference, and ** represents extremely significant difference.

The total antioxidant capacity of five edible fungi was enhanced under GSS (Figure 2). The total antioxidant capacity of *P. ostreatus* varied from 185.64% to 394.77%, showing significant increases in the P1, P3, P6, and P7 fruit bodies by 236, 104, 164, and 109%, respectively. *F. filiformis* exhibited a total antioxidant capacity between 126.02 and 229.65%, with the J1 and J7 fruit bodies increasing by 72.12 and 27.04%, respectively. *P. eryngii* total antioxidant capacity ranged from 98.15% to 278.44%, with the XB2, XB3, and XB4 strains experiencing increases of 51.96, 126.2, and 138.39%, respectively. *A. heimuer* showed a total antioxidant capacity between 107.38 and 189.88%, with a notable increase of 57.79% in the h4 strain. *A. cornea* demonstrated a total antioxidant capacity of 478.87 and 555.22%, with increases of 353.15 and 356.86% in the M2 and M6, respectively.

A significant enhancement in the antioxidant capacity of *P. eryngii*, grown on a novel substrate mixed with coffee grounds and thyme, was demonstrated in previous research, which was similar to the results of this study (40). Indirect evidence is provided by this result, showing that the use of ginger straw in the culture substrate positively affects the nutritional value of five edible fungi. The introduction of compounds with antioxidant properties is likely facilitated by the inclusion of ginger straw, which enhances the overall antioxidant capacity of the edible fungi, a change generally associated with increased nutritional value.

#### 3.2.3 The effect of GSS on hydrolyzed amino acids and protein quality

**Figure 3:**
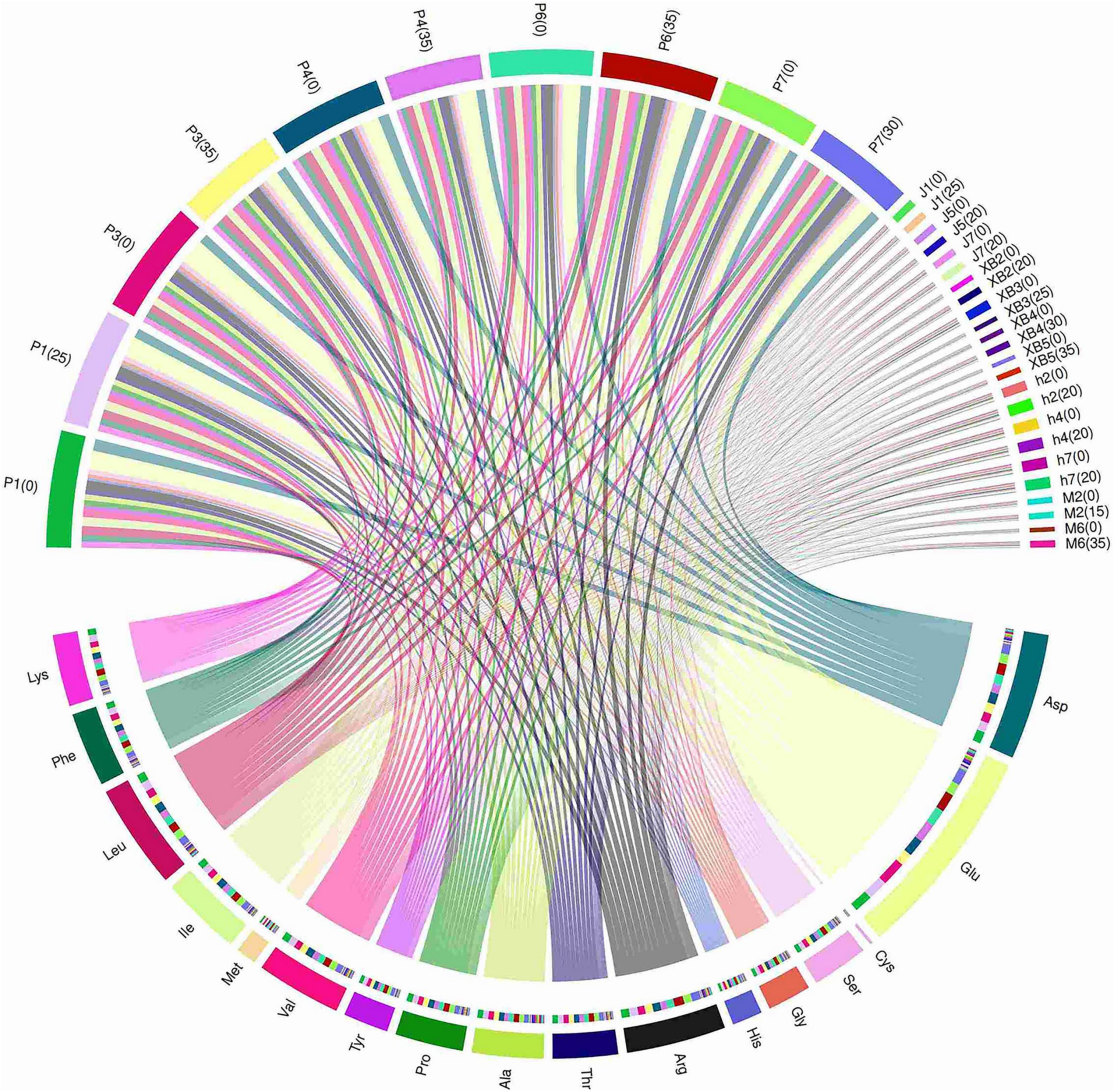
Circos analysis of hydrolyzed amino acid content of five edible fungi on the cottonseed hull substrate and GSS.

When the quality of dietary protein sources is discussed, the composition of amino acids is recognized as a core element due to its direct relation to the nutritional value and bioavailability of proteins. The protein quality of five edible fungi cultivated on a GSS was assessed (Table 4; Figure 3).

**Table 4:** Hydrolyzed amino acid content of five edible fungi on the cottonseed hull substrate and GSS (mg/g).

Group	Amino acid				
	TAA	EAA	NEAA	E/T	E/N
P1(0)	137.72 ± 0.34 b	53.46 ± 0.04 b	84.25 ± 0.36 b	0.39 ± 0.02 c	0.63 ± 0.03 c
P1(25)	139.67 ± 0.3 a	53.75 ± 0.01 ab	85.92 ± 0.3 a	0.38 ± 0.08 cd	0.63 ± 0.02 cd
P3(0)	133.03 ± 0.58 d	47.97 ± 0.12 ef	85.06 ± 0.7 ab	0.36 ± 0.09 g	0.56 ± 0.06 g
P3(35)	119.93 ± 0.71 f	48.94 ± 0.35 d	71 ± 0.43 f	0.41 ± 0.03 a	0.69 ± 0.04 a
P4(0)	135.74 ± 0.4 c	54.1 ± 0.1 a	81.63 ± 0.49 c	0.4 ± 0.01 b	0.66 ± 0.05 b
P4(35)	115.34 ± 0.29 h	42.39 ± 0.42 h	72.95 ± 0.21 e	0.37 ± 0.07 f	0.58 ± 0.07 f
P6(0)	122.72 ± 0.5 e	47.44 ± 0.19 f	75.28 ± 0.76 d	0.39 ± 0.07 cd	0.63 ± 0.09 cd
P6(35)	137.1 ± 0.5 bc	51.84 ± 0.13 c	85.28 ± 0.4 ab	0.38 ± 0.08 e	0.61 ± 0.02 e
P7(0)	117.7 ± 1.2 g	48.15 ± 0.24e	69.5.7 ± 1.1 g	0.41 ± 0.03 a	0.69 ± 0.09 a
P7(30)	118.94 ± 1.1 fg	45.58 ± 0.51 g	73.36 ± 0.54 e	0.38 ± 0.01 d	0.62 ± 0.04 d
J1(0)	8.79 ± 0.3 a	4.06 ± 0.17 a	4.73 ± 0.14 a	0.46 ± 0.01 a	0.86 ± 0.01 a
J1(25)	8.16 ± 0.14 b	3.7 ± 0.04 ab	4.5 ± 0.18 ab	0.45 ± 0.01 a	0.83 ± 0.04 a
J5(0)	8.01 ± 0.21 b	3.48 ± 0.12 b	4.5 ± 0.13 ab	0.44 ± 0.01 a	0.77 ± 0.01 a
J5(20)	8.03 ± 0.47 b	3.61 ± 0.31 b	4.43 ± 0.2 ab	0.45 ± 0.02 a	0.81 ± 0.05 a
J7(0)	7.92 ± 0.14 b	3.9 ± 0.13 ab	4.1 ± 0.41 b	0.49 ± 0.02 a	0.94 ± 0.09 a
J7(20)	8.54 ± 0.1 ab	4.04 ± 0.12 a	4.5 ± 0.18 ab	0.47 ± 0.02 a	0.9 ± 0.06 a
XB2(0)	6.09 ± 0.06 d	2.89 ± 0.06 cd	3.19 ± 0.05 d	0.48 ± 0.01 a	0.91 ± 0.03 ab
XB2(20)	7.74 ± 0.09 b	3.29 ± 0.03 b	4.44 ± 0.11 b	0.43 ± 0.01 c	0.74 ± 0.02 d
XB3(0)	10.82 ± 0.39 a	4.79 ± 0.21 a	6.03 ± 0.17 a	0.44 ± 0.01 bc	0.79 ± 0.01 cd
XB3(25)	5.22 ± 0.16 f	2.29 ± 0.11 e	2.92 ± 0.09 e	0.44 ± 0.01 bc	0.79 ± 0.04 cd
XB4(0)	5.73 ± 0.07 e	2.74 ± 0.09 d	2.99 ± 0.08 de	0.48 ± 0.01 a	0.92 ± 0.05 a
XB4(30)	7.49 ± 0.09 b	3.23 ± 0.02 b	4.26 ± 0.08 b	0.43 ± 0.01 c	0.76 ± 0.01 d
XB5(0)	4.83 ± 0.15 g	2.31 ± 0.17 e	2.51 ± 0.04 f	0.48 ± 0.02 a	0.92 ± 0.07 a
XB5(35)	6.59 ± 0.04 c	3.02 ± 0.02 bc	3.58 ± 0.01 c	0.46 ± 0.01 b	0.84 ± 0.01 bc
h2(0)	11.31 ± 0.12 c	4.58 ± 0.1 b	6.73 ± 0.04 b	0.4 ± 0.01 a	0.68 ± 0.01 a
h2(20)	13.05 ± 0.28 a	5.22 ± 0.03 a	7.83 ± 0.29 a	0.39 ± 0.01 a	0.67 ± 0.03 a
h4(0)	12.6 ± 0.2 ab	5.1 ± 0.15 a	7.59 ± 0.09 a	0.4 ± 0.01 a	0.67 ± 0.02 a
h4(20)	12.5 ± 0.3 ab	5.06 ± 0.14 a	7.68 ± 0.2a	0.4 ± 0.01a	0.66 ± 0.01a
h7(0)	12.7 ± 0.1 ab	5.06 ± 0.09 a	7.69 ± 0.06 a	0.4 ± 0.01 a	0.66 ± 0.01 a
h7(20)	12.4 ± 0.18 b	5.01 ± 0.09 a	7.43 ± 0.09 a	0.4 ± 0.01 a	0.67 ± 0.01 a
M2(0)	6.91 ± 0.49 b	3.04 ± 0.32 b	3.87 ± 0.17 b	0.44 ± 0.02 a	0.78 ± 0.05 a
M2(15)	7.26 ± 0.37 b	3.22 ± 0.18 b	4.04 ± 0.19 ab	0.44 ± 0.01 a	0.79 ± 0.01 a
M6(0)	5.31 ± 0.3 c	2.28 ± 0.04 c	3.03 ± 0.34 c	0.43 ± 0.03 a	0.76 ± 0.09 a
M6(35)	8.08 ± 0.26 a	3.69 ± 0.08 a	4.39 ± 0.23 a	0.46 ± 0.01 a	0.84 ± 0.04 a

Different lowercase letters indicate significant differences, *p* < 0.05. Group represents: strain name (ginger straw addition ratio, %).

Essential amino acid (EAA) is a key factor affecting protein quality, and 17 amino acids were identified in the five edible fungi ranging from 2.28 to 54.1 mg/g on the GSS. In addition, total amino acid (TAA) content was found to vary from 4.83 to 139.67 mg/g. TAA and EAA accumulation in the fruiting bodies was notably enhanced by the GSS. Specifically, the highest content of TAA and EAA in *P. ostreatus* were 115.34–139.67 mg/g and 42.39–53.75 mg/g, respectively. Compared to the control, TAA increases of 1.95 and 14.4 mg/g were displayed by P1 and P6, respectively; EAA increases of 0.97 and 4.4 mg/g were demonstrated by P3 and P6. Second, TAA content between 12.4 and 13.05 mg/g and EAA content of 5.01–5.22 mg/g were displayed by *A. heimuer*, with increases of 1.74 mg/g in TAA and 0.64 mg/g in EAA exhibited by h2. TAA content of 7.26 and 8.08 mg/g and EAA content of 3.22 and 3.68 mg/g were featured by M2 and M6 of *A. cornea*, respectively, with a notable increase by M6 of 2.77 mg/g in TAA and 1.41 mg/g in EAA. A TAA range of 5.22–7.74 mg/g and an EAA range of 2.29–3.29 mg/g were shown by *P. eryngii*, with significant TAA increases of 1.65, 1.76, and 1.76 mg/g registered by XB2, XB4, and XB5, and respective EAA increases of 0.4, 0.49, and 0.71 mg/g. Finally, the TAA and EAA contents of *F. filiformis* ranged from 4.83 to 10.82 mg/g and 2.2 to 4.79 mg/g. A significant decreasing trend in TAA and EAA contents was exhibited by *F. filiformis*, potentially attributed to varied nutrient absorption and metabolism from the GSS across the edible fungi. In the study by Wang et al., TAA and EAA contents of 126.7 and 347.5 mg/g, respectively, were exhibited by *P. ostreatus* grown on beer mash. Lower TAA and EAA contents were shown by *P. ostreatus* grown on the GSS compared to this experiment, likely attributed to the higher nitrogen and amino acid contents in beer mash that could enhance protein levels (41). Conversely, TAA and EAA contents ranging from 12.79 g/100 g to 19.09 g/100 g and 5.26 g/100 g to 7.81 g/100 g were found in fruiting bodies in Jin et al.’s study using herbal residue substrate, similar to those from ginger straw (42). It was suggested that substrates distinctly influence the protein content and composition of fruiting bodies, with ginger straw shown to have the potential to improve the quality of edible fungi proteins.

The E/T ratio in the five edible fungi grown on the GSS varied between 0.36 and 0.49, closely aligning with the 0.36 E/T ratio seen in *P. ostreatus* cultivated on beer mash (41). In addition, the E/N ratio between essential and non-essential amino acids in these edible fungi spanned from 0.56 to 0.94, similar to the 0.69 to 0.71 range found in edible fungus grown on corn cob substrate supplemented with traditional Chinese medicinal residues (42). The FAO/WHO recommends an ideal protein pattern with an E/T ratio of approximately 40% and an E/N ratio exceeding 60% for high-quality proteins. The five edible fungi in this study exhibit E/T and E/N ratios that align with these FAO/WHO criteria, demonstrating their excellence as protein sources in terms of both content and quality when grown on the GSS.

To assess the protein quality of edible fungi, amino acid score (AAS), chemical score (CS), and essential amino acid index (EAAI) were calculated. The AAS and CS measure the similarity of essential amino acid content in proteins to a standard reference; an AAS near 1 signifies high nutritional value, while a CS near 1 indicates superior amino acid composition. Conversely, AAS or CS values below 1 signify the presence of limiting amino acids, the lowest of which is identified as the first limiting amino acid. The EAAI comprehensively assesses protein quality by comparing the geometric mean of EAA ratios, thus categorizing proteins into different nutritional value levels. An EAAI ≤0.75 denotes unsuitability as a protein source; 0.75 ≤ EAAI ≤ 0.85 as suitable; 0.85 ≤ EAAI ≤ 0.95 as good; and EAAI ≥ 0.95 as high-quality.

The amino acid composition of edible fungi on ginger straw was assessed using the AAS. Methionine and cysteine were identified as the primary limiting amino acids, as detailed in Table 5. Despite variations, AAS scores for all strains and the control group were found to be above 1. It was indicated that edible fungi grown on ginger straw possess high amino acid nutritional values, which enhance digestion and absorption. CS analysis indicated that methionine and cysteine were the primary limiting amino acids, aligning with the AAS results (Table 6). Meanwhile, the protein quality was assessed using the EAAI, indicating that all five edible fungi grown on ginger straw had EAAI values exceeding 0.95, highlighting their potential as high-quality protein sources (Table 6). Notably, the EAAI was used to assess the protein quality of edible fungi, introducing an innovative perspective and methodology to the field.

**Table 5:** The amino acid score (AAS) of five edible fungi on the cottonseed hull substrate and GSS.

Group	Amino acid							
	Thr	Val	Met+Cys	Ile	Leu	Phe + Tyr	Lys	Total
P1(0)	1.2 ± 0.02 bc	1.31 ± 0.02 c	0.54 ± 0.01b	1.8 ± 0.01 bc	2.34 ± 0.02c	1.52 ± 0.01 c	1.03 ± 0.01 c	1.22 ± 0.01 c
P1(25)	1.21 ± 0.01 de	1.27 ± 0.03 de	0.54 ± 0.01 b	1.79 ± 0.03 bc	2.27 ± 0.03de	1.53 ± 0.01 bc	1.07 ± 0.01 b	1.21 ± 0.01 d
P3(0)	1.2 ± 0.01 bc	1.16 ± 0.02 f	0.5 ± 0.02 c	1.67 ± 0.02 d	2.14 ± 0.01 g	1.48 ± 0.01 d	0.94 ± 0.01 e	1.14 ± 0.01 h
P3(35)	1.29 ± 0.03 a	1.39 ± 0.01 b	0.53 ± 0.01bc	1.92 ± 0.04 a	2.43 ± 0.02 a	1.58 ± 0.01 a	1.06 ± 0.02 b	1.27 ± 0.01 a
P4(0)	1.2 ± 0.01 de	1.37 ± 0.02 b	0.59 ± 0.02 a	1.91 ± 0.06 a	2.4 ± 0.01 b	1.59 ± 0.01 a	1.03 ± 0.02 c	1.25 ± 0.01 b
P4(35)	1.16 ± 0.02 f	1.27 ± 0.02 de	0.45 ± 0.03 d	1.81 ± 0.02 b	2.21 ± 0.02 f	1.45 ± 0.01 e	0.87 ± 0.01 f	1.15 ± 0.01 g
P6(0)	1.21 ± 0.02 cd	1.26 ± 0.01 de	0.53 ± 0.02 b	1.76 ± 0.02 c	2.28 ± 0.01 d	1.53 ± 0.01 bc	1.11 ± 0.01 a	1.21 ± 0.01 cd
P6(35)	1.21 ± 0.02 de	1.25 ± 0.02 e	0.51 ± 0.01bc	1.76 ± 0.02 c	2.24 ± 0.02 ef	1.53 ± 0.01 bc	1.03 ± 0.02 c	1.19 ± 0.01 f
P7(0)	1.27 ± 0.02 ab	1.43 ± 0.02 a	0.52 ± 0.01bc	1.93 ± 0.03 a	2.42 ± 0.01 ab	1.59 ± 0.02 a	1.03 ± 0.01 c	1.27 ± 0.01 a
P7(30)	1.18 ± 0.01 ef	1.29 ± 0.02 cd	0.52 ± 0.01bc	1.84 ± 0.03 b	2.27 ± 0.02 de	1.54 ± 0.02 b	0.98 ± 0.02 d	1.2 ± 0.01 e
J1(0)	1.55 ± 0.15 ab	1.48 ± 0.03 ab	0.4 ± 0.04 b	1.88 ± 0.24 ab	1.23 ± 0.08 a	1.57 ± 0.02 ab	1.19 ± 0.1 ab	1.32 ± 0.01abc
J1(25)	1.27 ± 0.2 b	1.58 ± 0.05 ab	0.37 ± 0.03 b	1.93 ± 0.09 ab	1.38 ± 0.05 a	1.33 ± 0.26 b	1.24 ± 0.05 ab	1.3 ± 0.03 bc
J5(0)	1.14 ± 0.28 b	1.61 ± 0.14 a	0.78 ± 0.36 a	1.71 ± 0.02 b	0.87 ± 0.3 b	1.67 ± 0.16 a	1.14 ± 0.08 bc	1.24 ± 0.02 c
J5(20)	1.22 ± 0.08 b	1.56 ± 0.02 ab	0.55 ± 0.1 ab	1.86 ± 0.05 ab	1.28 ± 0.06 a	1.62 ± 0.07 a	0.94 ± 0.16 c	1.28 ± 0.05 bc
J7(0)	1.74 ± 0.34 a	1.44 ± 0.07 b	0.65 ± 0.22ab	2.02 ± 0.19 a	1.18 ± 0.08 a	1.53 ± 0.05 ab	1.39 ± 0.18 a	1.38 ± 0.07 a
J7(20)	1.49 ± 0.04 ab	1.45 ± 0.06 b	0.49 ± 0.1 ab	1.85 ± 0.1 ab	1.24 ± 0.03 a	1.79 ± 0.13 a	1.21 ± 0.07 ab	1.35 ± 0.04 ab
XB2(0)	1.17 ± 0.11 a	1.67 ± 0.08 b	0.55 ± 0.14 a	2.23 ± 0.27 ab	1.28 ± 0.09 a	1.76 ± 0.02 ab	0.99 ± 0.1 abc	1.36 ± 0.02 a
XB2(20)	1.11 ± 0.05 a	1.34 ± 0.06 d	0.46 ± 0.1 a	1.74 ± 0.19 c	1.12 ± 0.05 d	1.75 ± 0.17 ab	1.03 ± 0.02 ab	1.22 ± 0.02 c
XB3(0)	1.22 ± 0.06 a	1.61 ± 0.08 bc	0.46 ± 0.05 a	1.92 ± 0.05 bc	1.16 ± 0.04bcd	1.65 ± 0.07 b	0.93 ± 0.03 abc	1.27 ± 0.01 bc
XB3(25)	1.01 ± 0.11 a	1.51 ± 0.11 c	0.54 ± 0.21 a	2.06 ± 0.25 abc	1.22 ± 0.06abcd	1.73 ± 0.01 ab	0.83 ± 0.04 bc	1.26 ± 0.04 bc
XB4(0)	1.28 ± 0.32 a	1.71 ± 0.06 b	0.56 ± 0.23 a	2.29 ± 0.16 a	1.25 ± 0.04 abc	1.81 ± 0.12 ab	0.85 ± 0.19 bc	1.36 ± 0.04 a
XB4(30)	1.11 ± 0.04 a	1.47 ± 0.07 cd	0.47 ± 0.06 a	1.79 ± 0.06 c	1.15 ± 0.05 cd	1.59 ± 0.15 b	1.08 ± 0.12 a	1.23 ± 0.01 c
XB5(0)	1.18 ± 0.07 a	1.84 ± 0.11 a	0.53 ± 0.05 a	2.24 ± 0.21 ab	1.27 ± 0.06 ab	1.88 ± 0.14 a	0.8 ± 0.14 c	1.37 ± 0.05 a
XB5(35)	1.21 ± 0.23 a	1.61 ± 0.06 bc	0.59 ± 0.16 a	2.03 ± 0.09 abc	1.19 ± 0.09 abcd	1.69 ± 0.05 ab	0.99 ± 0.07 abc	1.31 ± 0.01 bc
h2(0)	1.68 ± 0.04 ab	1.29 ± 0.03 a	0.26 ± 0.03 a	1.17 ± 0.18 a	1.33 ± 0.06 a	1.39 ± 0.11 a	0.86 ± 0.08 a	1.16 ± 0.01 a
h2(20)	1.69 ± 0.09 a	1.33 ± 0.11 a	0.39 ± 0.09 a	1.26 ± 0.08 a	1.24 ± 0.05 a	1.31 ± 0.07 a	0.82 ± 0.09 a	1.14 ± 0.03 a
h4(0)	1.64 ± 0.07 ab	1.24 ± 0.05 a	0.36 ± 0.08 a	1.28 ± 0.07 a	1.29 ± 0.04 a	1.3 ± 0.05 a	0.91 ± 0.13 a	1.15 ± 0.02 a
h4(20)	1.49 ± 0.07 b	1.17 ± 0.08 a	0.33 ± 0.04 a	1.25 ± 0.06 a	1.28 ± 0.02 a	1.34 ± 0.05 a	1.01 ± 0.04 a	1.13 ± 0.01 a
h7(0)	1.59 ± 0.07 ab	1.27 ± 0.05 a	0.35 ± 0.03 a	1.23 ± 0.05 a	1.29 ± 0.02 a	1.37 ± 0.1 a	0.8 ± 0.06 a	1.13 ± 0.01 a
h7(20)	1.64 ± 0.07 ab	1.25 ± 0.05 a	0.39 ± 0.08 a	1.25 ± 0.05 a	1.27 ± 0.02 a	1.43 ± 0.04 a	0.8 ± 0.06 a	1.15 ± 0.01 a
M2(0)	1.31 ± 0.02 a	1.52 ± 0.07 a	0.46 ± 0.11 a	1.24 ± 0.46 a	1.27 ± 0.04 a	1.87 ± 0.07 a	0.99 ± 0.08 a	1.26 ± 0.04 a
M2(15)	1.42 ± 0.06 a	1.49 ± 0.06 a	0.48 ± 0.19 a	1.4 ± 0.15 a	1.2 ± 0.07 a	1.93 ± 0.04 a	1.09 ± 0.25 a	1.27 ± 0.01 a
M6(0)	1.32 ± 0.23 a	1.44 ± 0.21 a	0.42 ± 0.02 a	1.28 ± 0.39 a	1.19 ± 0.14 a	1.96 ± 0.61 a	0.87 ± 0.1 a	1.23 ± 0.08 a
M6(35)	1.42 ± 0.05 a	1.58 ± 0.08 a	0.39 ± 0.07 a	1.56 ± 0.11 a	1.3 ± 0.01 a	1.83 ± 0.1 a	0.97 ± 0.01 a	1.3 ± 0.04 a

Different lowercase letters indicate significant differences, *p* < 0.05. Group represents: strain name (ginger straw addition ratio, %).

**Table 6:** The chemical score (CS) and essential amino acid index (EAAI) of five edible fungi on the cottonseed hull substrate and GSS.

Group	Amino acid								
	Thr	Val	Met+Cys	Ile	Leu	Phe + Tyr	Lys	Total	EAAI
P1(0)	0.97 ± 0.01 bc	0.89 ± 0.01 c	0.34 ± 0.01 b	1.14 ± 0.02 bc	0.93 ± 0.01 c	0.91 ± 0.01 c	0.89 ± 0.01 c	0.86 ± 0.01 c	0.99 ± 0.01 b
P1(25)	0.95 ± 0.01 de	0.87 ± 0.02de	0.34 ± 0.01 b	1.14 ± 0.01 bc	0.91 ± 0.02 de	0.92 ± 0.02 bc	0.92 ± 0.01 b	0.85 ± 0.01 d	0.99 ± 0.01 bc
P3(0)	0.98 ± 0.01 bc	0.8 ± 0.02 f	0.32 ± 0.02 c	1.06 ± 0.02 d	0.85 ± 0.01 g	0.87 ± 0.01 d	0.81 ± 0.01 e	0.81 ± 0.01 h	0.98 ± 0.01 d
P3(35)	1.01 ± 0.02 a	0.95 ± 0.01 b	0.34 ± 0.01 bc	1.22 ± 0.01 a	0.97 ± 0.02 a	0.95 ± 0.01 a	0.91 ± 0.02 b	0.89 ± 0.02 a	0.99 ± 0.01 a
P4(0)	0.94 ± 0.01 de	0.93 ± 0.01 b	0.37 ± 0.02 a	1.22 ± 0.01 a	0.95 ± 0.01 b	0.95 ± 0.01 a	0.88 ± 0.02 c	0.88 ± 0.02 b	0.98 ± 0.01 d
P4(35)	0.91 ± 0.02 f	0.87 ± 0.01 de	0.28 ± 0.02 d	1.15 ± 0.02 b	0.88 ± 0.01 f	0.87 ± 0.01 e	0.77 ± 0.01 f	0.81 ± 0.03 g	0.99 ± 0.01 a
P6(0)	0.95 ± 0.01 cd	0.86 ± 0.01 de	0.34 ± 0.01 b	1.12 ± 0.01 c	0.91 ± 0.01 d	0.92 ± 0.02 bc	0.95 ± 0.01 a	0.85 ± 0.03 cd	0.99 ± 0.01 bc
P6(35)	0.95 ± 0.01 de	0.86 ± 0.01 e	0.32 ± 0.02 bc	1.12 ± 0.01 c	0.89 ± 0.01 ef	0.92 ± 0.02 bc	0.89 ± 0.01 c	0.84 ± 0.01 f	0.98 ± 0.01 c
P7(0)	0.99 ± 0.01 ab	0.98 ± 0.02 a	0.33 ± 0.02 bc	1.23 ± 0.02 a	0.96 ± 0.01 ab	0.96 ± 0.01 a	0.88 ± 0.02 c	0.89 ± 0.01 a	0.99 ± 0.01 a
P7(30)	0.93 ± 0.01 ef	0.88 ± 0.01 cd	0.33 ± 0.01 bc	1.17 ± 0.01 b	0.91 ± 0.02 de	0.93 ± 0.01 b	0.84 ± 0.01 d	0.85 ± 0.01 e	0.99 ± 0.01 ab
J1(0)	1.21 ± 0.12 ab	1.01 ± 0.02 ab	0.26 ± 0.03 b	1.19 ± 0.16 ab	0.98 ± 0.06 a	0.94 ± 0.02 ab	1.02 ± 0.09 ab	0.93 ± 0.01 abc	1.33 ± 0.01 abc
J1(25)	1 ± 0.16 b	1.08 ± 0.03 ab	0.23 ± 0.02 b	1.23 ± 0.06 ab	1.1 ± 0.04 a	0.8 ± 0.16 b	1.07 ± 0.04 ab	0.92 ± 0.02 bc	1.33 ± 0.01 bc
J5(0)	0.89 ± 0.22 b	1.1 ± 0.09 a	0.5 ± 0.23 a	1.09 ± 0.01 b	0.69 ± 0.24 b	1 ± 0.1 a	0.98 ± 0.01 bc	0.88 ± 0.02 c	1.32 ± 0.01 c
J5(20)	0.96 ± 0.07 b	1.07 ± 0.01 ab	0.35 ± 0.06 ab	1.19 ± 0.03 ab	1.02 ± 0.01 a	0.97 ± 0.04 a	0.81 ± 0.14 c	0.9 ± 0.03 bc	1.33 ± 0.01 bc
J7(0)	1.36 ± 0.27 a	0.99 ± 0.05 b	0.41 ± 14 ab	1.29 ± 0.12 a	0.94 ± 0.07 a	0.82 ± 0.03 ab	1.2 ± 0.16 a	0.97 ± 0.05 a	1.35 ± 0.01 a
J7(20)	1.17 ± 0.03 ab	0.1 ± 0.04 b	0.31 ± 0.06 ab	1.18 ± 0.06 ab	0.98 ± 0.02 a	1.1 ± 0.08 a	1.04 ± 0.07 ab	0.95 ± 0.04 ab	1.34 ± 0.01 ab
XB2(0)	0.92 ± 0.09 a	1.15 ± 0.05 b	0.35 ± 0.09 a	1.4 ± 0.17 ab	1.02 ± 0.07 a	1. ± 0.01 ab	0.86 ± 0.1 abc	0.96 ± 0.02 a	1.34 ± 0.01 a
XB2(20)	0.87 ± 0.04 a	0.92 ± 0.05 d	0.3 ± 0.06 a	1.1 ± 0.12 c	0.89 ± 0.04 d	1.05 ± 0.1 ab	0.89 ± 0.01 ab	0.86 ± 0.02 c	1.32 ± 0.01 c
XB3(0)	0.96 ± 0.05 a	1.11 ± 0.05 bc	0.29 ± 0.03 a	1.22 ± 0.03 bc	0.92 ± 0.03 bcd	0.99 ± 0.04 b	0.8 ± 0.02 abc	0.9 ± 0.01 bc	1.33 ± 0.01 bc
XB3(25)	0.79 ± 0.08 a	1.04 ± 0.07 c	0.34 ± 0.13 a	1.31 ± 0.16 abc	0.97 ± 0.05abcd	1.04 ± 0.01 ab	0.71 ± 0.03 bc	0.88 ± 0.03 bc	1.33 ± 0.01 c
XB4(0)	1.01 ± 0.25 a	1.17 ± 0.04 b	0.36 ± 0.15 a	1.46 ± 0.1 a	1.01 ± 0.03 abc	1.08 ± 0.07 ab	0.73 ± 0.16 bc	0.96 ± 0.03 a	1.34 ± 0.01 a
XB4(30)	0.87 ± 0.03 a	1.01 ± 0.04 cd	0.3 ± 0.04 a	1.14 ± 0.04 cd	0.92 ± 0.04 cd	0.95 ± 0.09 b	0.93 ± 0.1 a	0.87 ± 0.01 c	1.32 ± 0.01 c
XB5(0)	0.92 ± 0.05 a	1.26 ± 0.07 a	0.34 ± 0.03 a	1.43 ± 0.13 ab	1.01 ± 0.05 ab	1.13 ± 0.08 a	0.69 ± 0.12 c	0.96 ± 0.04 a	1.34 ± 0.01 a
XB5(35)	0.94 ± 0.18 a	1.1 ± 0.04 bc	0.38 ± 0.1 a	1.29 ± 0.05 abc	0.95 ± 0.07 abcd	1.02 ± 0.03 ab	0.85 ± 0.06 abc	0.92 ± 0.01 bc	1.33 ± 0.01 ab
h2(0)	1.32 ± 0.03 ab	0.89 ± 0.02 a	0.17 ± 0.02 a	0.75 ± 0.11 a	1.06 ± 0.04 a	0.83 ± 0.07 a	0.74 ± 0.07 a	0.81 ± 0.01 a	1.31 ± 0.01 a
h2(20)	1.33 ± 0.07 a	0.91 ± 0.07 a	0.25 ± 0.06 a	0.8 ± 0.05 a	0.98 ± 0.04 a	0.78 ± 0.04 a	0.7 ± 0.08 a	0.8 ± 0.02 a	1.31 ± 0.01 a
h4(0)	1.3 ± 0.05 ab	0.85 ± 0.03 a	0.23 ± 0.05 a	0.82 ± 0.05 a	1.03 ± 0.03 a	0.78 ± 0.03 a	0.78 ± 0.11 a	0.81 ± 0.01 a	1.31 ± 0.01 a
h4(20)	1.17 ± 0.06 b	0.8 ± 0.06 a	0.21 ± 0.02 a	0.8 ± 0.04 a	1.02 ± 0.01 a	0.81 ± 0.03 a	0.89 ± 0.04 a	0.8 ± 0.01 a	1.31 ± 0.01 a
h7(0)	1.25 ± 0.05 ab	0.87 ± 0.03 a	0.22 ± 0.02 a	0.78 ± 0.03 a	1.03 ± 0.02 a	0.82 ± 0.06 a	0.69 ± 0.05 a	0.8 ± 0.01 a	1.31 ± 0.01 a
h7(20)	1.29 ± 0.05 ab	0.86 ± 0.04 a	0.25 ± 0.05 a	0.79 ± 0.03 a	1.01 ± 0.02 a	0.86 ± 0.02 a	0.69 ± 0.05 a	0.81 ± 0.01 a	1.31 ± 0.01 a
M2(0)	1.03 ± 0.02 a	1.04 ± 0.05 a	0.29 ± 0.07 a	0.79 ± 0.29 a	1.01 ± 0.03 a	1.12 ± 0.04 a	0.86 ± 0.07 a	0.88 ± 0.03 a	1.32 ± 0.01 a
M2(15)	1.11 ± 0.05 a	1.02 ± 0.04 a	0.3 ± 0.12 a	0.89 ± 0.07 a	0.95 ± 0.06 a	1.04 ± 0.02 a	0.94 ± 0.22 a	0.89 ± 0.01 a	1.32 ± 0.01 a
M6(0)	1.04 ± 0.18 a	0.99 ± 0.15 a	0.27 ± 0.02 a	0.81 ± 0.25 a	0.95 ± 0.12 a	1.18 ± 0.37 a	0.74 ± 0.09 a	0.87 ± 0.06 a	1.32 ± 0.01 a
M6(35)	1.11 ± 0.04 a	1.08 ± 0.05 a	0.25 ± 0.05 a	0.77 ± 0.07 a	1.03 ± 0.01 a	1.1 ± 0.06 a	0.83 ± 0.01 a	0.92 ± 0.03 a	1.33 ± 0.01 a

Different lowercase letters indicate significant differences, *p* < 0.05. Group represents: strain name (ginger straw addition ratio, %).

#### 3.2.4 The effect of GSS on the flavor-contributing amino acids

**Figure 4:**
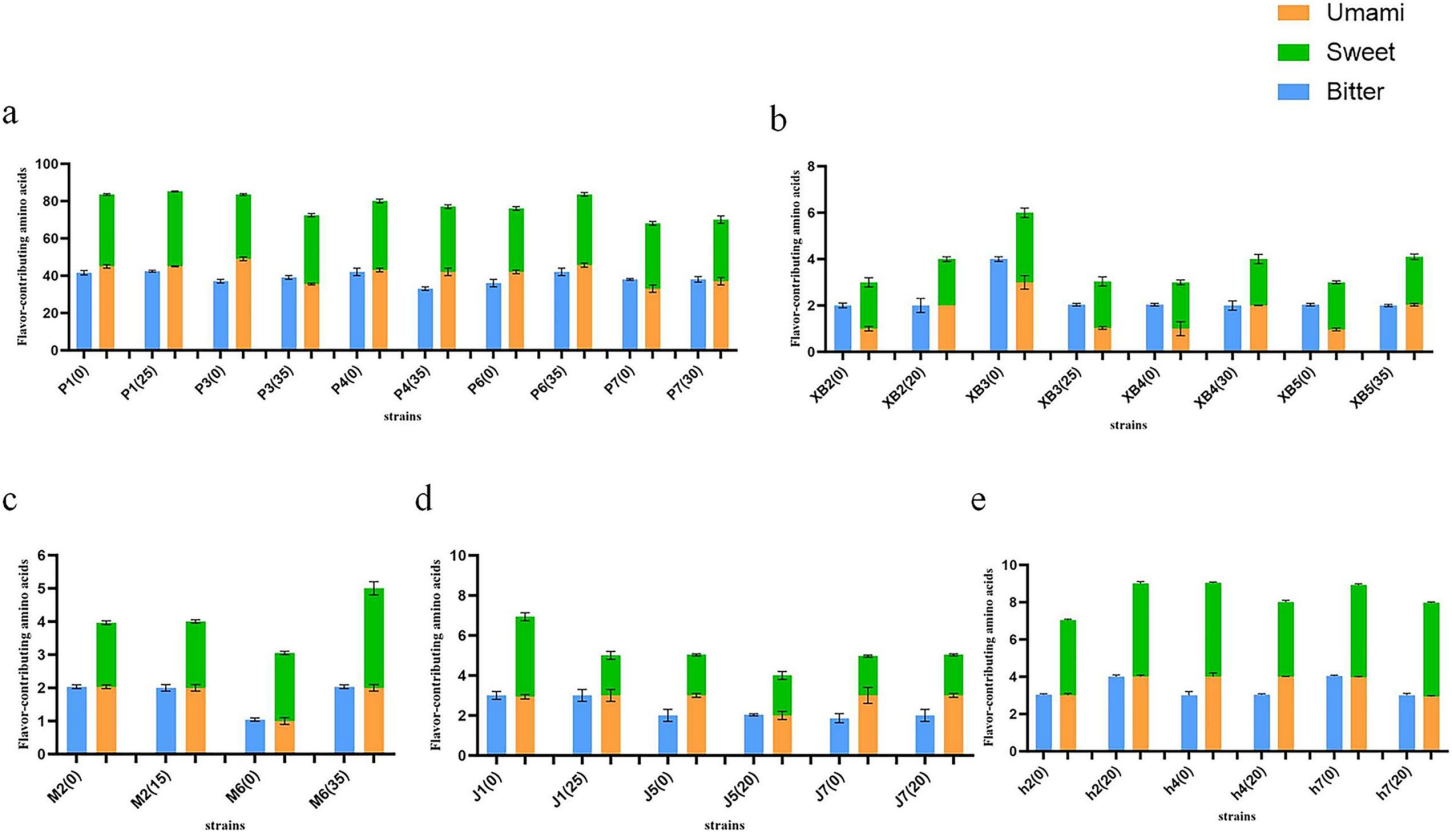
Content of flavor-contributing amino acids of five edible fungi on the cottonseed hull substrate and GSS (mg/g). **(A)** Content of flavor-contributing amino acids of *Pleurotus ostreatus*. **(B)** Content of flavor-contributing amino acids of *Pleurotus eryngii*. **(C)** Content of flavor-contributing amino acids of *Auricularia cornea*. **(D)** Content of flavor-contributing amino acids of *Flammulina filiformis*. **(E)** Content of flavor-contributing amino acids of *Auricularia heimuer*.

The flavor of fruiting bodies is correlated with the presence of free amino acids, which are known to contribute distinct taste sensations based on their side chains. Lysine, glutamic acid, and aspartic acid are classified as umami amino acids, while glycine, alanine, serine, threonine, proline, and histidine are classified as sweet amino acids. Valine, leucine, isoleucine, methionine, and arginine are categorized as bitter amino acids. The content of flavor-contributing amino acids in five edible fungi was quantified (Figure 4).

The results showed that the content of umami and sweet amino acids in the five edible fungi was generally increased. The highest comprehensive content of umami and sweet amino acid levels in *P. ostreatus* was found to range from 70 to 84.18 mg/g, with increases being recorded as 0.71, 6.82, and 1.4 mg/g for strains P1, P6, and P7, respectively. Second, combined umami and sweet amino acid levels in *A. heimuer* were displayed from 7.95 to 8.34 mg/g, with increases noted in strains h2 and h4 by 1.15 and 0.06 mg/g, respectively. Combined umami and sweet amino acid levels in *P. eryngii* were noted to be between 2.86 and 4.57 mg/g, with increases recorded as 1.33, 1.25, and 1 mg/g for strains XB2, XB4, and XB5, respectively. In *F. filiformis*, combined umami and sweet amino acids were observed to range from 4.76 to 5.12 mg/g, with an increase of 0.32 mg/g being recorded in strain J7. Finally, combined umami and sweet amino acid levels in *A. cornea* were recorded at 4.37 and 4.77 mg/g, with increases of 0.21 and 1.55 mg/g observed. Across all cases, combined levels of umami and sweet amino acids were found to exceed those of bitter amino acids. From a biochemical perspective, the beneficial effects of ginger straw on enhancing the flavor and taste of edible fungi were confirmed by this discovery, which also introduces a new strategy for improving edible fungi quality. The fermentation liquid of *Lentinula Edodes* was used to cultivate *F. filiformis*, resulting in umami amino acid levels between 1.015 and 1.287 mg/g, sweet levels between 0.934 and 1.707 mg/g, and bitter levels between 1.002 and 1.327 mg/g (43). *P. ostreatus* were cultivated by Koutrotsios et al. on substrates composed of grape marc, wheat straw, and olive mill byproducts, achieving umami amino acid levels from 6.27 to 8.94 mg/g, sweet from 9.75 to 18.17 mg/g, and bitter from 17.29 to 30.09 mg/g (44). Amino acid levels in edible fungi grown on these substrates were found to be lower than those grown on ginger straw, illustrating the beneficial impact of ginger straw on the flavor and taste of edible fungi.

#### 3.2.5 The effect of GSS on trace element content

**Figure 5:**
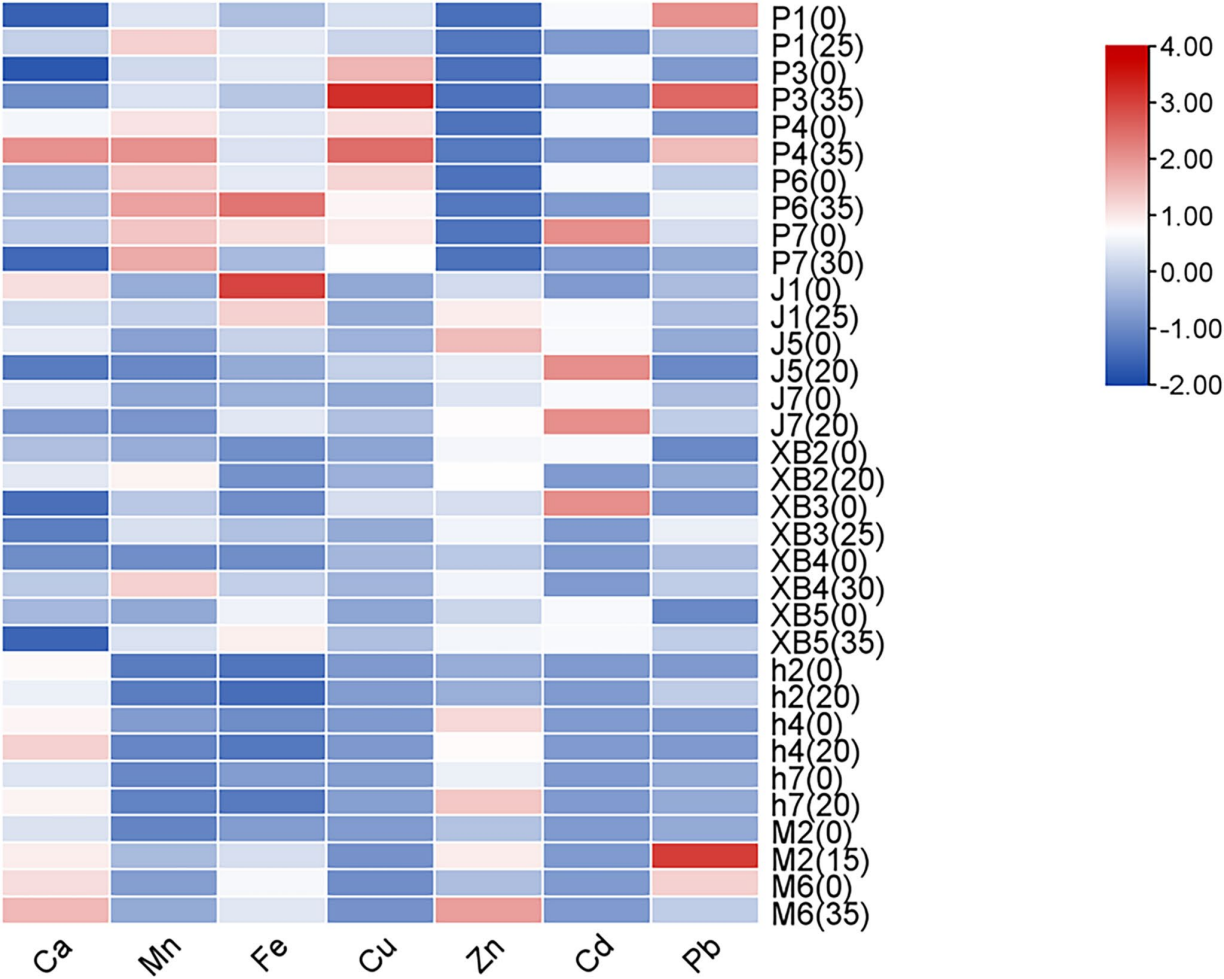
Heat map analysis of trace element content of five edible fungi on the cottonseed hull substrate and GSS.

Minerals are considered crucial for the biological activities of organisms, being fundamental components of cellular structures and functions, and are involved in various biochemical reactions and physiological processes. Owing to their unique growth modes and biological characteristics, mineral elements are absorbed and accumulated more effectively by edible fungi from the environment. Consequently, mineral content was considered a primary indicator of edible fungi quality (45). The accumulation of minerals in the production of edible fungi was investigated by measuring the mineral content in the five edible fungi (Figure 5).

Despite significant variations, calcium (Ca) has been found to be the predominant element in all edible fungi, followed by manganese (Mn), iron (Fe), and zinc (Zn). In the study by Zhou et al., *P. eryngii* cultivated on corn stalks were shown to contain Ca levels ranging from 66.9 to 110.0 mg/kg, Mg from 5.78 to 6.92 mg/kg, Fe from 28.5 to 40.1 mg/kg, copper (Cu) from 2.73 to 3.91 mg/kg, and Zn from 59.9 to 81.9 mg/kg. When compared to ginger straw, the contents of all minerals, except Zn, were observed to be lower or similar (18). Similarly, levels of Cu, Fe, and Zn in *A. heimuer*, cultivated on corn stalks by Yao et al., were found to parallel those in ginger straw (37). It has been further confirmed by this evidence that edible fungi were a reliable source of minerals. In addition, the Cd and Pb levels in these fruiting bodies remained low, adhering to the national standard GB2762-2022, “Maximum levels of contaminants in foods.”

It was indicated by subsequent analysis that the manganese (Mn) and Zn contents of five edible fungi were significantly increased by the GSS compared to the control. In *P. ostreatus*, Mn content was found to vary from 6.47 to 10.15 mg/kg, with increases being noted across strains P1, P3, P4, P6, and P7 by 2, 0.25, 2.08, 1.08, and 0.69 mg/kg, respectively. Zn content in *P. ostreatus* was recorded to span from 6.84 to 9.53 mg/kg, with increases being observed in strains P1, P3, P4, and P6 by 2.92, 0.31, 2.21, and 1.7 mg/kg, respectively. Zn content in *P. ostreatus* was recorded to span from 6.84 to 9.53 mg/kg, with increases being observed in strains P1, P3, P4, and P6 by 2.92, 0.31, 2.21, and 1.7 mg/kg, respectively. Mn content in *P. eryngii* was shown to range from 6.43 to 8.55 mg/kg, with increases recorded in strains XB2, XB4, and XB5 by 2.89, 4.74, and 1.81 mg/kg, respectively. Zn content ranged from 53.75 to 57.21 mg/kg, with increases observed in strains XB3, XB4, and XB5 by 7.82, 15.73, and 11.8 mg/kg, respectively. Zn levels in *A. heimuer* were presented to range from 28.64 to 73.41 mg/kg and Mn levels from 3.27 to 3.56 mg/kg. An increase in Zn content by 21.13 mg/kg was notably observed in strain h7, while Mn content was found to decrease. Mn content in *A. cornea* was exhibited to be 5.22 and 4.71 mg/kg in strains M2 and M6, respectively, with an increase of 1.71 mg/kg being observed in strain M2. Zn content was recorded at 62.70 and 85.18 mg/kg, with increases of 26.83 and 51.06 mg/kg, respectively.

The Ca and Cu levels of five edible fungi were markedly increased by the GSS. In *P. ostreatus*, Ca levels ranging from 112.46 mg/kg to 578.32 mg/kg were observed, with increases being recorded in strains P1, P3, and P4 by 216.99, 106.14, and 187.89 mg/kg, respectively. Meanwhile, Cu levels from 5.36 to 16.96 mg/kg were noted, with increases in strains P3 and P4 by 6.06 and 5.1 mg/kg. In *P. eryngii*, Ca levels were from 105.14 to 363.72 mg/kg, with increases recorded in strains XB2, XB3, and XB4 by 81.02, 28.59, and 119.3 mg/kg. Cu levels ranging from 2.98 to 4.11 mg/kg were noted, with increases in strains XB2 and XB5 by 0.65 and 1.41 mg/kg. Ca levels in *A. heimuer* were shown to range from 378.18 to 476.5 mg/kg, with increases being noted in strains h4 and h7 by 55.17 and 68.6 mg/kg. Ca levels in *A. cornea* were exhibited at 433.29 and 515.57 mg/kg, with increases recorded by 80.76 and 56.83 mg/kg. Cu content was found to be 1.62 mg/kg, with an increase noted in strain M6 by 0.22 mg/kg. In *F. filiformis*, Cu levels were found to range from 2.98 to 5.07 mg/kg; however, Cu absorption was inhibited by the GSS, resulting in significant reductions of 122, 217, and 150 mg/kg.

In addition, the influence of GSS on the Fe content across five edible fungi exhibited no consistent trend. In *P. ostreatus*, Fe levels varied from 46.53 to 106.6 mg/kg, with increases noted in strains P1 and P6 by 14.17 and 44.08 mg/kg, respectively, and decreases in strains P4 and P7. *F. filiformis* showed Fe levels from 41.42 to 81.66 mg/kg, with decreases in strains J1 and J5 by 38.02 and 13.26 mg/kg, respectively, and an increase in strain J7 by 18.81 mg/kg. *P. eryngii* exhibited Fe content from 33.1 to 73.81 mg/kg, with increases in strains XB3, XB4, and XB5 by 16.92, 21.99, and 8.42 mg/kg, respectively. *A. heimuer* presented Fe levels from 21.12 to 24.97 mg/kg, with reductions in strains h4 and h7 by 6.97 and 11.78 mg/kg, respectively. *A. cornea*, strains M2 and M6, displayed Fe levels of 58.92 and 61.69 mg/kg, with an increase of 22.2 mg/kg in M2 and a decrease of 5.55 mg/kg in M6.

### 3.3 The effect of GSS on the comprehensive quality of five edible fungi

The membership function method aims to transform fuzzy concepts into precise values and provide rigorous support for decision-making. By setting a threshold, the degree of membership of things is quantified (0–1), with close to 1 indicating high membership and close to 0 indicating low membership. This process improves the accuracy, scientificity, and rationality of the evaluation system (46, 47).

**Figure 6:**
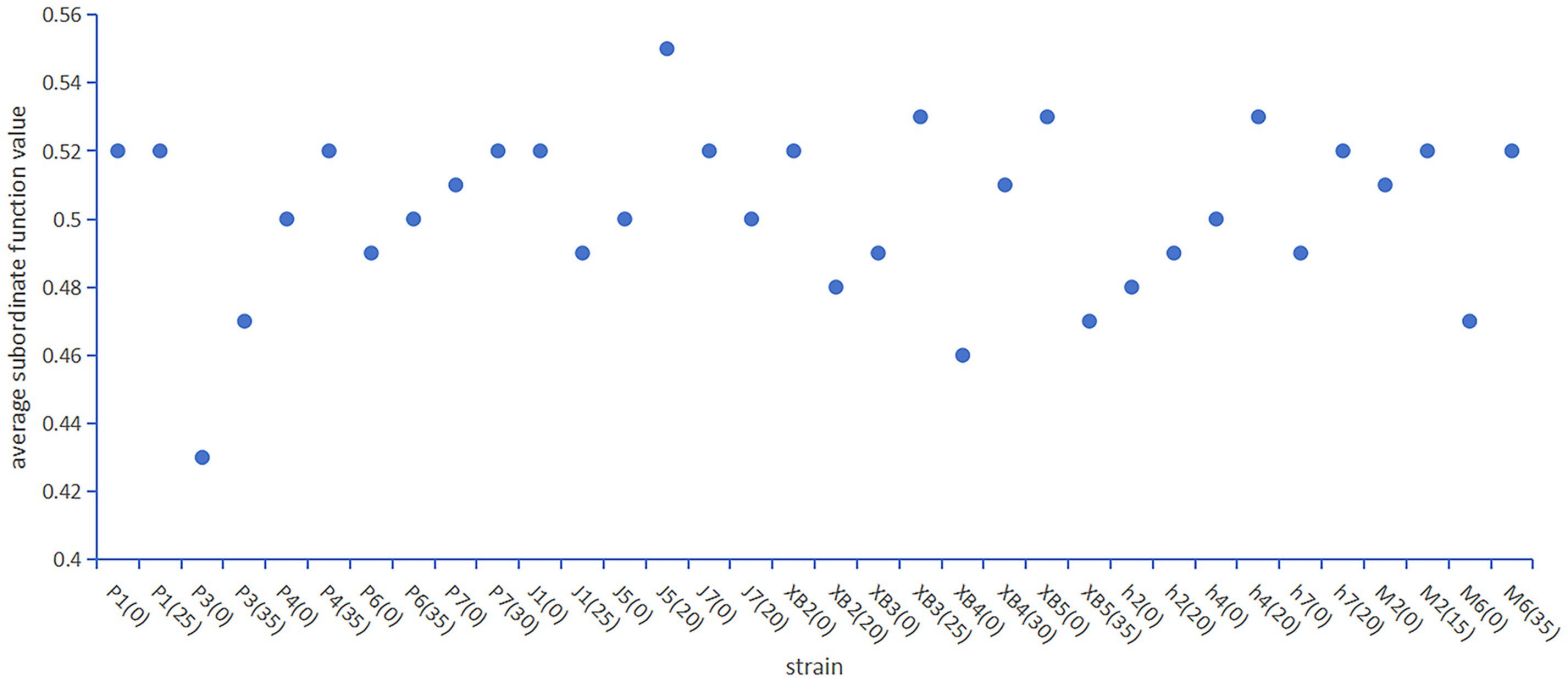
The average subordinate function value of five edible fungi on the cottonseed hull substrate and GSS.

Given the effectiveness of the membership function method in quality assessment, as demonstrated by the evaluation of 14 commercial edible fungus varieties by Wang et al. (48). The method was employed in this study to thoroughly and systematically assess the impact of the GSS on the characteristics of five edible fungi (Figure 6).

The results showed that the average subordinate function value of the ginger straw culture substrate of *P. ostreatus*, *A. heimuers,* and *A. cornea* was higher than that of the control group. Specifically, the average subordinate function value was 0.52, 0.47, 0.52, 0.50, and 0.52 for P1, P3, P4, P6, and P7 of *P. ostreatus*; 0.49, 0.53, and 0.52 for h2, h4, and h7 of *A. heimuers*; and 0.52 for both M2 and M6 of *A. cornea*, respectively. As for *F. filiformis* and *P. eryngii*, differences in the average subordinate function value were observed between different strains. In *F. filiformis*, average subordinate function values of 0.49 and 0.50 were shown by strains J1 and J7, respectively, being 0.03 and 0.02 below the control group. The average subordinate function value of 0.55, which was 0.05 above the control group, was exhibited by strain J5 of *F. filiformis*. The average subordinate function values of 0.48 and 0.47, 0.04 and 0.06 below the control group, were recorded by strains XB2 and XB5 of *P. eryngii*. Average subordinate function values of 0.53 and 0.51, 0.04 and 0.02 above the control group, were recorded by strains XB3 and XB4 of *P. eryngii*. The results suggested that the comprehensive quality of P1, P3, P4, P6, and P7 of *P. ostreatus*, J5 of *F. filiformis*, XB3 and XB4 of *P. eryngii*, h2, h4, and h7 of *A. heimuer,* and M2 and M6 of *A. cornea* were positively affected by GSS. However, significant improvement in the comprehensive quality of J1 and J7 of *F. filiformis* and XB2 and XB5 of *P. eryngii* were not observed with the use of GSS.

## 4 Conclusion

In summary, GSS is suitable for the cultivation of five major edible fungi. Especially, the average subordinate function value of P1, P3, P4, P6, and P7 of *P. ostreatus*, J5 of *F. filiformis*, XB3 and XB4 of *P. eryngii*, h2, h4, and h7 of *A. heimuer,* and M2 and M6 of *A. cornea* on the GSS was higher than that of conventional (cottonseed hull) substrate, indicating that the comprehensive quality of these strains was improved by the GSS. The BE of five edible fungi cultivated on the GSS ranged from 61.69% to 147.59%, confirming the effectiveness of GSS as a culture substrate. In addition, a significant increase in crude protein, reducing sugar, crude fiber, total antioxidant capacity, and mineral levels was observed in five edible fungi cultivated on the GSS. The flavor profile was positively influenced by an increase in the combined content of umami and sweet amino acids attributable to the GSS. Notably, both TAA and EAA contents of five edible fungi were increased significantly on the GSS. The AAS and CS of five edible fungi were found to approach 1, with an EAAI above 0.95, suggesting an improvement in protein quality.

In recent years, the production of edible fungi has increased significantly, and the demand for cultivation substrate materials has increased rapidly. The supply of traditional materials such as sawdust and cottonseed shells is limited, while new materials such as beer grains, coffee grounds, and nut shells emerge. These new materials come from a wide range of sources, are low cost, and are mostly common waste, which is both environmentally friendly and can reduce cultivation costs.

This study proves the suitability of a new cultivation substrate, ginger straw, for cultivating edible fungi. This substrate is characterized by its abundant resource availability and low cost while demonstrating a notable enhancement in the yield, fruiting body traits, and nutritional properties of the edible fungi. These findings provide a theoretical foundation for the cultivation of edible fungi using ginger straw and serve as a commendable demonstration for exploring novel raw materials for the cultivation of edible fungi.

## Data Availability

The original contributions presented in the study are included in the article/Supplementary material, further inquiries can be directed to the corresponding authors.

## Footnotes

^1^ www.graphpad.com
