# Supplementary material for: Enhancing the yield, fruiting body traits, and nutritional properties of five major edible fungi through the exploitation of ginger straw substrate
Source: Front Nutr. 2025 May 16;12:1583716. doi: 10.3389/fnut.2025.1583716 (PMC12122320; doi:10.3389/fnut.2025.1583716)
Supplement: Supplementary file 1 [file Table_1.docx]

# Screening of strains and determination of ginger straw addition ratio of five major edible fungi

# 1. Screening of excellent strains

In order to promote the follow-up experimental research, we extensively collected five top seven edible fungi from China, including *Pleurotus ostreatus*, *Flammulina filiformis*, *Pleurotus eryngii*, *Auricularia heimuer* and *Auricularia cornea*, with a total of 33 strains. Then, we used Potato Dextrose Agar (PDA) substrate and conventional cottonseed hull culture substrate to determine the growth rate and growth situation of these five edible fungi. The purpose of this process is to investigate the efficiency of direct nutrient absorption and the ability of different strains to decompose the cultivated substrate. Based on the comprehensive evaluation of these two indicators, we further screened out the strains with excellent performance.

## 1.1 Evaluation of the growth performance of strains on the PDA substrate

## 1.1.1 Evaluation of the growth performance of *P. ostreatus* strains on the PDA substrate

The growth rates and performances of *P. ostreatus* strains on PDA substrate were measured. The results were found to reveal significant differences in growth rates among the strains, as shown in Table S1. The mycelial growth rates of the seven *P. ostreatus* strains were found to range from 4.61 mm/d to 5.76 mm/d. Strain P3 was identified as exhibiting the fastest growth rate at 5.76 mm/d, whereas slower growth rates of 4.61 mm/d and 4.58 mm/d were observed for strains P2 and P5, respectively. Regarding growth performance, strains P1, P3, P4, P6, and P7 were classified as excellent, while strains P2 and P5 were classified as good.

Table S1 The growth performance of *Pleurotus ostreatus* strains on PDA substrate.

| Strains | Growth rate（mm/d） | Color | Marginal  regularity | Density | Growth |
| --- | --- | --- | --- | --- | --- |
| P1 | 5.26±0.13 bc | White | neat | bushy | +++ |
| P2 | 4.61±0.17 d | White | neat | sparse | ++ |
| P3 | 5.76±0.1 a | White | neat | bushy | +++ |
| P4 | 5.36±0.11 bc | White | neat | bushy | +++ |
| P5 | 5.36±0.11 bc | White | Untidy | bushy | ++ |
| P6 | 4.95±0.68 b | White | neat | bushy | +++ |
| P7 | 5.36±0.12 bc | White | neat | bushy | +++ |

Note:“+” poor growth; “++” growth is average; “+++” the growth is better；Different lowercase letters indicate significant differences, *p*<0.05.

### 1.1.2 Evaluation of the growth performance of *F. filiformis* strains on the PDA substrate

Significant differences in growth rates among various strains of *F. filiformis* on PDA substrate were observed, as shown in Table S2. Among the 11 strains of *F. filiformis*, strain J5 was found to exhibit the fastest growth rate of 4.84 mm/d, whereas strain J6 was observed to display the slowest growth rate of 0.56 mm/d. Differences in growth performance among the strains were also noted. Strains J1, J5, and J7 were classified as excellent, whereas strains J2, J3, J6, J8, J9, and J10 were classified as good. Strains J4 and J11 were classified as average.

Table S2 The growth performance of *Flammulina filiformis* strains on PDA substrate.

| Strains | Growth rate（mm/d） | Color | Marginal  regularity | Density | Growth |
| --- | --- | --- | --- | --- | --- |
| J1 | 4.33±0.17 b | White | neat | bushy | +++ |
| J2 | 3.23±0.06 f | White | Relatively neat | bushy | ++ |
| J3 | 3.35±0.08 ef | ashen | neat | bushy | ++ |
| J4 | 2.87±0.02 g | White | Relatively neat | sparse | + |
| J5 | 4.84±0.07 a | White | neat | bushy | +++ |
| J6 | 0.56±0.07 h | White | neat | sparse | ++ |
| J7 | 4.42±0.06 b | White | neat | bushy | +++ |
| J8 | 3.45±0.03 e | ashen | neat | bushy | ++ |
| J9 | 3.64±0.08 d | ashen | Relatively neat | bushy | ++ |
| J10 | 3.91±0.13 c | ashen | Untidy | Denser | ++ |
| J11 | 3.68±0.07 d | ashen | neat | Denser | + |

Note:“+” poor growth; “++” growth is average; “+++” the growth is better；Different lowercase letters indicate significant differences, *p*<0.05.

### 1.1.3 Evaluation of the growth performance of *A. heimuer* strains on the PDA substrate

Significant differences in growth rates among various strains of *A. heimuer* were noted, as illustrated in Table S3. The growth rates of the three strains were found to range from 1.24 mm/d to 2.55 mm/d. Strain h5 was identified as exhibiting the slowest growth rate of 1.24 mm/d, whereas strain h2 was recognized as displaying the fastest growth rate of 2.55 mm/d. Regarding mycelial growth performance, strains h2, h4, and h7 were classified as excellent, whereas strains h1, h3, and h6 were classified as good. Strain h5 was classified as average regarding mycelial growth performance.

Table S3 The growth performance of *Auricularia heimuer* strains on PDA substrate.

| Strains | Growth rate（mm/d） | Color | Marginal  regularity | Density | Growth |
| --- | --- | --- | --- | --- | --- |
| h1 | 2.24±0.04cd | snow white | Relatively neat | bushy | ++ |
| h2 | 2.55±0.02a | snow white | neat | bushy | +++ |
| h3 | 1.62±0.1e | White | neat | Denser | ++ |
| h4 | 2.42±0.01ab | snow white | neat | bushy | +++ |
| h5 | 1.24±0.06f | White | Relatively neat | Denser | + |
| h6 | 2.1±0.03d | snow white | Relatively neat | bushy | ++ |
| h7 | 2.34±0.13bc | snow white | neat | bushy | +++ |

Note:“+” poor growth; “++” growth is average; “+++” the growth is better；Different lowercase letters indicate significant differences, *p*<0.05.

## 1.1.4 Evaluation of the growth performance of *A. cornea* strains on the PDA substrate

The growth rates and performance of various strains of *A. cornea* on PDA substrate were evaluated, as presented in Table S4 The growth rates of the strains were found to range from 2.11 mm/d to 3.9 mm/d, with strain M3 being identified as exhibiting the slowest growth rate of 2.11 mm/d, while strain M2 was recognized as exhibiting the fastest growth rate of 3.9 mm/d. Differences in growth performance ratings among the strains were noted, with strains M2 and M6 being classified as excellent, while strains M1 and M3 were classified as good.

Table S4 The growth performance of *Auricularia cornea* strains on PDA substrate.

| Strains | Growth rate（mm/d） | Color | Marginal  regularity | Density | Growth |
| --- | --- | --- | --- | --- | --- |
| M1 | 2.58±0.14b | snow white | Relatively neat | bushy | ++ |
| M2 | 3.9±0.12a | snow white | neat | bushy | +++ |
| M3 | 2.11±0.33c | snow white | Relatively neat | bushy | ++ |
| M6 | 3.82±0.09a | snow white | neat | bushy | +++ |

Note:“+” poor growth; “++” growth is average; “+++” the growth is better；Different lowercase letters indicate significant differences, *p*<0.05.

### 1.1.5 Evaluation of the growth performance of *P. eryngii* strains on the PDA substrate

The mycelial growth rates of various strains of *P. eryngii* on PDA substrate were not found to be significantly different, as shown in Table S5. The growth rates of the strains were recorded to range from 3.20 mm/d to 3.27 mm/d. Regarding mycelial growth performance, no differences were noted among the strains of *P. eryngii*, all of which were characterized by a white, dense appearance with smooth edges and classified as excellent.

Table S5 The growth performance of *Pleurotus eryngii* strains on PDA substrate.

| Strains | Growth rate（mm/d） | Color | Marginal  regularity | Density | Growth |
| --- | --- | --- | --- | --- | --- |
| XB2 | 3.25±0.03a | white | neat | bushy | +++ |
| XB3 | 3.27±0.01a | white | neat | bushy | +++ |
| XB4 | 3.2±0.03a | white | neat | bushy | +++ |
| XB5 | 3.21±0.05a | white | neat | bushy | +++ |

Note:“+” poor growth; “++” growth is average; “+++” the growth is better；Different lowercase letters indicate significant differences, *p*<0.05.

## 1.2 Evaluation of the growth performance of strains on conventional cottonseed hull culture substrate

### 1.2.1 Evaluation of the growth performance of *P. ostreatus* strains on the conventional cottonseed hull culture substrate

The growth rates of *P. ostreatus* strains on conventional cottonseed hull culture substrate were found to vary from 3.65 to 4.19 mm/d (Table S6). The fastest-growing strain, P6, was identified with a growth rate of 4.16 mm/d, whereas the slowest strain, P2, was identified with a rate of 3.65 mm/d. Significant differences in mycelial growth among the strains were noted; P1, P3, P4, P6, and P7 were classified as excellent, while P2 and P5 were classified as good.

Table S6 The growth performance of *Pleurotus ostreatus* in conventional cottonseed hull culture substrate.

| Strains | Growth rate（mm/d） | Color | Marginal  regularity | Density | Growth |
| --- | --- | --- | --- | --- | --- |
| P1 | 4.14±0.11a | snow white | neat | bushy | +++ |
| P2 | 3.65±0.12b | snow white | neat | Denser | ++ |
| P3 | 4.19±0.04a | snow white | neat | bushy | +++ |
| P4 | 4.13±0.12a | snow white | neat | bushy | +++ |
| P5 | 3.66±0.05b | snow white | Relatively neat | bushy | ++ |
| P6 | 4.16±0.19a | snow white | neat | bushy | +++ |
| P7 | 4.1±0.19a | snow white | neat | bushy | +++ |

Note:“+” poor growth; “++” growth is average; “+++” the growth is better；Different lowercase letters indicate significant differences, *p*<0.05.

### 1.2.2 Evaluation of the growth performance of *F. filiformis* strains on the conventional cottonseed hull culture substrate

The growth rates of various strains of *F. filiformis* on conventional cottonseed hull culture substrate were found to vary from 2.91 to 4.45 mm/d (Table S7). The strain with the highest growth rate was identified as J7, which exhibited a rate of 4.45 mm/d, while the strain with the lowest rate was identified as J6, at 2.91 mm/d. Differences in the mycelial growth evaluation levels among the strains were noted: J1, J5, and J7 were classified as excellent, while J2, J3, J6, J8, J10, and J11 were classified as good, and J4 and J9 were classified as average.

Table S7 The growth performance of *Flammulina filiformis* in conventional cottonseed hull culture substrate.

| Strains | Growth rate（mm/d） | Color | Marginal  regularity | Density | Growth |
| --- | --- | --- | --- | --- | --- |
| J1 | 4.18±0.15 a | snow white | neat | bushy | +++ |
| J2 | 3.33±0.27 b | snow white | Relatively neat | bushy | ++ |
| J3 | 3.41±0.07 cd | snow white | Relatively neat | bushy | ++ |
| J4 | 3.24±0.08 d | snow white | Relatively neat | sparse | + |
| J5 | 4.33±0.15 a | snow white | neat | bushy | +++ |
| J6 | 2.91±0.08 e | snow white | Relatively neat | bushy | ++ |
| J7 | 4.45±0.11 a | snow white | neat | bushy | +++ |
| J8 | 3.44±0.33 cd | snow white | neat | Denser | ++ |
| J9 | 3.51±0.12 cd | snow white | Relatively neat | Denser | + |
| J10 | 3.62±0.09 bc | snow white | neat | Denser | ++ |
| J11 | 3.49±0.14 cd | snow white | neat | Denser | ++ |

Note:“+” poor growth; “++” growth is average; “+++” the growth is better；Different lowercase letters indicate significant differences, *p*<0.05.

### 1.2.3 Evaluation of the growth performance of *A. heimuer* strains on the conventional cottonseed hull culture substrate

The mycelial growth rates of various strains of *A. heimuer* on conventional cottonseed hull culture substrate were found to vary from 1.17 to 1.75 mm/d, with significant differences noted among the strains (Table S8). The strain exhibiting the highest growth rate was identified as h2, at 1.75 mm/d, whereas the strain with the lowest rate was identified as h5, at 1.17 mm/d. Differences in mycelial growth among the strains were observed: h2, h4, and h7 were classified as excellent, while h1, h3, h5, and h6 were classified as good.

Table S8 The growth performance of *Auricularia heimuer* in conventional cottonseed hull culture substrate.

| Strains | Growth rate（mm/d） | Color | Marginal  regularity | Density | Growth |
| --- | --- | --- | --- | --- | --- |
| h1 | 1.52±0.04 c | snow white | Untidy | bushy | ++ |
| h2 | 1.75±0.06 a | snow white | neat | bushy | +++ |
| h3 | 1.35±0.09 d | snow white | Relatively neat | bushy | ++ |
| h4 | 1.69±0.06 ab | snow white | neat | bushy | +++ |
| h5 | 1.17±0.01 e | snow white | Untidy | bushy | ++ |
| h6 | 1.49±0.06 c | snow white | Untidy | bushy | ++ |
| h7 | 1.61±0.05 bc | snow white | neat | bushy | +++ |

Note:“+” poor growth; “++” growth is average; “+++” the growth is better；Different lowercase letters indicate significant differences, *p*<0.05.

### 1.2.4 Evaluation of the growth performance of *A. cornea* strains on the conventional cottonseed hull culture substrate

The mycelial growth rates of various strains of *A. cornea* were found to range from 1.89 to 2.25 mm/d (Table S9), with the M2 strain being identified as having the highest growth rate at 2.24 mm/d, while the M3 strain was identified as having the lowest rate at 1.89 mm/d. Furthermore, differences in mycelial growth among the strains were noted: M2 and M6 were classified as excellent, while M1 and M3 were classified as good.

Table S9 The growth performance of *Auricularia cornea* in conventional cottonseed hull culture substrate.

| Strains | Growth rate（mm/d） | Color | Marginal  regularity | Density | Growth |
| --- | --- | --- | --- | --- | --- |
| M1 | 1.89±0.07 b | snow white | Untidy | bushy | ++ |
| M2 | 2.24±0.07 a | snow white | Relatively neat | bushy | +++ |
| M3 | 1.89±0.05 b | snow white | neat | bushy | ++ |
| M6 | 2.25±0.08 a | snow white | Relatively neat | bushy | +++ |

Note:“+” poor growth; “++” growth is average; “+++” the growth is better；Different lowercase letters indicate significant differences, *p*<0.05.

### 1.2.5 Evaluation of the growth performance of *P. eryngii* strains on the conventional cottonseed hull culture substrate

The mycelial growth rates of various strains of *P. eryngii* were found to range from 2.88 to 2.91 mm/d (Table S10), with the XB4 strain being identified as exhibiting the highest growth rate of 2.91 mm/d, while the XB3 strain was identified as having the lowest rate of 2.88 mm/d. No significant differences were observed in the growth evaluation levels among the strains, all of which were classified as excellent.

Table S10 The growth performance of *Pleurotus eryngii* in conventional cottonseed hull culture substrate.

| Strains | Growth rate（mm/d） | Color | Marginal  regularity | Density | Growth |
| --- | --- | --- | --- | --- | --- |
| XB2 | 2.9±0.12 a | snow white | neat | bushy | +++ |
| XB3 | 2.88±0.08 a | snow white | neat | bushy | +++ |
| XB4 | 2.91±0.07 a | snow white | neat | bushy | +++ |
| XB5 | 2.89±0.07 a | snow white | neat | bushy | +++ |

Note:“+” poor growth; “++” growth is average; “+++” the growth is better；Different lowercase letters indicate significant differences, *p*<0.05.

A comprehensive analysis of the growth rates and performance of various strains on PDA substrate and conventional cottonseed hull culture substrate was conducted, resulting in the selection of excellent strains: *P. ostreatus* (P1, P3, P4, P6, P7), *F. filiformis* (J1, J5, J7), *A. heimuer* (h2, h4, h7), *A. cornea* (M2, M6), and *P. eryngii* (XB2, XB3, XB4, XB5). These strains will be employed in subsequent experiments.

# 2 Determination of ginger straw addition ratio of five edible fungi

To preliminarily determine the addition ratio of ginger straw, formulation optimization experiments were performed based on Table S11. Numerous test tubes were filled to measure mycelial growth rates and performance, and the formulations for different strains in ginger straw substrate were selected according to the results. A comprehensive analysis was conducted to evaluate the growth rates, performance, and utilization of ginger straw in the substrate for different strains, aiming to determine the appropriate addition ratios. The final ginger straw addition ratios for each strain were established as follows: for *P. ostreatus*, the P1 strain was found to utilize a substrate with a 25% ginger straw addition, while the P3, P4, and P6 strains utilized a 35% addition, and the P7 strain utilized a 30% addition. For *F. filiformis*, the J1 and J5 strains were found to utilize a 25% addition, while the J7 strain utilized a 20% addition. For *P. eryngii*, the XB2 strain utilized a 20% addition, the XB3 strain utilized 25%, the XB4 strain utilized 30%, and the XB5 strain utilized 35%. For *A. heimuer*, all test strains utilized a 20% addition, whereas for *A. cornea*, the M2 strain utilized a 15% addition and the M6 strain utilized a 35% addition.

Table S11 Culture substrate formula

| 名称  Name | 配方  Formulations | 条件  Conditions |
| --- | --- | --- |
| 1. *Ostreatus* Cultivation substrate Formula Using Ginger Straw (Mass Fraction) | Recipe 1: 10% ginger straw, 80% cottonseed hull, 8% wheat bran, 2% quicklime  Recipe 2: 15% ginger straw, 75% cottonseed hull, 8% wheat bran, 2% quicklime  Recipe 3: 20% ginger straw, 60% cottonseed hull, 8% wheat bran, 2% quicklime  Recipe 4: 25% ginger straw, 65% cottonseed hull, 8% wheat bran, 2% quicklime  Recipe 5: 30% ginger straw, 60% cottonseed hull, 8% wheat bran, 2% quicklime  Recipe 6: 35% ginger straw, 55% cottonseed hull, 8% wheat bran, 2% quicklime  Recipe 7: 40% ginger straw, 50% cottonseed hulls, 8% wheat bran, 2% quicklime | Autoclaving（121℃，  0.12~0.14 MPa，3 h） |
| *F. filiformis* Cultivation substrate Formula Using Ginger Straw (Mass Fraction) | Recipe 1: 10% ginger straw, 77% cottonseed hull, 11% wheat bran, 2% quicklime  Recipe 2: 15% ginger straw, 72% cottonseed hull, 11% wheat bran, 2% quicklime  Recipe 3: 20% ginger straw, 67% cottonseed hull, 11% wheat bran, 2% quicklime  Recipe 4: 25% ginger straw, 62% cottonseed hull, 11% wheat bran, 2% quicklime  Recipe 5: 30% ginger straw, 57% cottonseed hull, 11% wheat bran, 2% quicklime  Recipe 6: 35% ginger straw, 52% cottonseed hull, 11% wheat bran, 2% quicklime  Recipe 7: 40% ginger straw, 47% cottonseed hull, 11% wheat bran, 2% quicklime | Autoclaving（121℃，  0.12~0.14 MPa，3 h） |
| *P. eryngii* Cultivation substrate Formula Using Ginger Straw (Mass Fraction) | Recipe 1: 10% ginger straw, 83% cottonseed hull, 6% wheat bran, 1% quicklime  Recipe 2: 15% ginger straw, 78% cottonseed hull, 6% wheat bran, 1% quicklime  Recipe 3: 20% ginger straw, 73% cottonseed hull, 6% wheat bran, 1% quicklime  Recipe 4: 25% ginger straw, 68% cottonseed hull, 6% wheat bran, 1% quicklime  Recipe 5: 30% ginger straw, 63% cottonseed hulls, 6% wheat bran, 1% quicklime  Recipe 6: 35% ginger straw, 58% cottonseed hull, 6% wheat bran, 1% quicklime  Recipe 7: 40% ginger straw, 53% cottonseed hull, 6% wheat bran, 1% quicklime | Autoclaving（121℃，  0.12~0.14 MPa，3 h） |
| 1. *polytricha* Cultivation substrate Formula Using Ginger Straw (Mass Fraction) | Recipe 1: 10% ginger straw, 68% cottonseed hull, 20% wheat bran, 3% quicklime, 1% superphosphate, 1% light calcium powder  Recipe 2: 15% ginger straw, 63% cottonseed hull, 20% wheat bran, 3% quicklime, 1% superphosphate, 1% light calcium powder  Recipe 3: 20% ginger straw, 58% cottonseed hull, 20% wheat bran, 3% quicklime, 1% superphosphate, 1% light calcium powder  Recipe 4: 25% ginger straw, 53% cottonseed hull, 20% wheat bran, 3% quicklime, 1% superphosphate, 1% light calcium powder  Recipe 5: 30% ginger straw, 48% cottonseed hull, 20% wheat bran, 3% quicklime, 1% superphosphate, 1% light calcium powder  Recipe 6: 35% ginger straw, 43% cottonseed hull, 20% wheat bran, 3% quicklime, 1% superphosphate, 1% light calcium powder  Recipe 7: 40% ginger straw, 38% cottonseed hull, 20% wheat bran, 3% quicklime, 1% superphosphate, 1% light calcium powder | Autoclaving（121℃，  0.12~0.14 MPa，3 h） |
| *A. heimuer* Cultivation substrate Formula Using Ginger Straw (Mass Fraction) | Recipe 1: 10% ginger straw, 68% cottonseed hull, 20% wheat bran, 1% gypsum, 1% sucrose  Recipe 2: 15% ginger straw, 63% cottonseed hull, 20% wheat bran, 1% gypsum, 1% sucrose  Recipe 3: 20% ginger straw, 58% cottonseed hull, 20% wheat bran, 1% gypsum, 1% sucrose  Recipe 4: 25% ginger straw, 53% cottonseed hull, 20% wheat bran, 1% gypsum, 1% sucrose  Recipe 5: 30% ginger straw, 48% cottonseed hull, 20% wheat bran, 1% gypsum, 1% sucrose  Recipe 6: 35% ginger straw, 43% cottonseed hull, 20% wheat bran, 1% gypsum, 1% sucrose  Recipe 7: 40% ginger straw, 38% cottonseed hull, 20% wheat bran, 1% gypsum, 1% sucrose | Autoclaving（121℃，  0.12~0.14 MPa，3 h） |

The specific results are as follows:

## 2.1 The determination of optimal ginger straw addition ratios for the *P. ostreatus* strains

The growth rates and performance of *P. ostreatus* strains were analyzed using the conventional cottonseed hull culture substrate as a control, with ginger straw added at ratios of 10%, 15%, 20%, 25%, 30%, 35%, and 40%, as presented in Table S12. Faster mycelial growth rates were exhibited by strain P1 in substrate containing ginger straw at 15%, 20%, and 25%, with growth rates of 8.02 mm/d, 8.13 mm/d, and 8.04 mm/d, respectively, compared to the control group. In substrate containing ginger straw at ratios of 10%, 15%, 20%, and 25%, the growth performance was rated as excellent, consistent with that of the control group.

The growth rate of strain P3 was measured to range from 6.53 to 7.52 mm/d, with no significant differences observed across substrate containing varying proportions of ginger straw, all of which exceeded the mycelial growth rate of the control group. The growth performance of strain P3 was rated as excellent with ginger straw additions of 25%, 30%, and 35%, which was consistent with the control group.

A growth rate of strain P4 was exhibited in the range of 6.79 to 7.71 mm/d, with the fastest rate recorded at a ginger straw addition of 35%, which surpassed the mycelial growth rate of the control group. The mycelial growth performance of strain P4 was found to vary across substrate with different proportions of ginger straw, receiving excellent ratings at the 25%, 30%, and 35% addition levels, consistent with the control group.

A growth rate ranging from 6.6 to 7.23 mm/d was exhibited by the P6 strain in ginger straw culture substrate, with the highest growth being observed in the control group. The mycelial growth of the P6 strain was found to vary across different substrate and was rated excellent when ginger straw was added at a 35% ratio, aligning with the results of the control group.

The highest growth rate in the control group was achieved by the P7 strain, reaching 7.22 mm/d. The mycelial growth of the P7 strain was evaluated to vary with different ratios of ginger straw, receiving an excellent rating at both 25% and 30%, thereby outperforming the control group.

Table S12 The growth performance of *Pleurotus ostreatus* strains in different proportion of ginger straw substrate.

| Strains | Addition ratio (%) | Growth rate（mm/d） | Color | Marginal regularity | Density | Growth |
| --- | --- | --- | --- | --- | --- | --- |
| P1 | 0 | 7.87±0.05 ab | snow white | neat | bushy | +++ |
|  | 10 | 7.67±0.21 abc | snow white | neat | bushy | +++ |
|  | 15 | 8.02±0.32 a | snow white | neat | bushy | +++ |
|  | 20 | 8.13±0.36 a | snow white | neat | bushy | +++ |
|  | 25 | 8.04±0.24 abc | snow white | neat | bushy | +++ |
|  | 30 | 7.29±0.12 c | snow white | neat | bushy | ++ |
|  | 35 | 7.37±0.26 bc | snow white | Relatively neat | bushy | ++ |
|  | 40 | 6.6±0.08 d | snow white | Relatively neat | Denser | ++ |
| P3 | 0 | 6.85±0.37ab | snow white | neat | bushy | +++ |
|  | 10 | 6.93±0.33 ab | snow white | Relatively neat | bushy | ++ |
|  | 15 | 7.05±0.17 ab | snow white | Relatively neat | bushy | ++ |
|  | 20 | 7.52±0.09 ab | snow white | Relatively neat | bushy | ++ |
|  | 25 | 7.25±0.53 ab | snow white | neat | bushy | +++ |
|  | 30 | 7.48±0.39 ab | snow white | neat | bushy | +++ |
|  | 35 | 7.25±0.18 ab | snow white | neat | bushy | +++ |
|  | 40 | 6.53±0.07 b | white | Relatively neat | bushy | ++ |
| P4 | 0 | 7.56±0.22 ab | snow white | neat | bushy | +++ |
|  | 10 | 7.56±0.11 ab | snow white | Relatively neat | bushy | ++ |
|  | 15 | 7.2±0.13 cd | snow white | Relatively neat | bushy | ++ |
|  | 20 | 7.14±0.13 d | snow white | Relatively neat | bushy | ++ |
|  | 25 | 6.79±0.19 bcd | snow white | neat | bushy | +++ |
|  | 30 | 7.35±0.38 abc | snow white | neat | bushy | +++ |
|  | 35 | 7.71±0.16 a | snow white | neat | bushy | +++ |
|  | 40 | 7.24±0.16 bcd | snow white | Relatively neat | bushy | ++ |
| P6 | 0 | 7.91±0.08 a | snow white | neat | bushy | +++ |
|  | 10 | 7.15±0.36 bc | snow white | Untidy | bushy | ++ |
|  | 15 | 6.87±0.14 bcd | snow white | Untidy | Denser | ++ |
|  | 20 | 6.95±0.26 bcd | snow white | Untidy | Denser | ++ |
|  | 25 | 6.79±3.37 cd | snow white | Untidy | Denser | ++ |
|  | 30 | 7.23±0.06 b | snow white | Untidy | bushy | ++ |
|  | 35 | 7.13±0.19 bc | snow white | neat | bushy | +++ |
|  | 40 | 6.6±0.06 d | snow white | neat | bushy | ++ |
| P7 | 0 | 7.22±0.15 a | snow white | Relatively neat | bushy | ++ |
|  | 10 | 6.74±0.05 bc | snow white | Untidy | bushy | ++ |
|  | 15 | 6.61±0.09 c | snow white | Untidy | bushy | ++ |
|  | 20 | 6.94±0.09 ab | white | Untidy | bushy | ++ |
|  | 25 | 6.82±0.24 bc | white | neat | bushy | +++ |
|  | 30 | 7.01±0.18 ab | snow white | neat | bushy | +++ |
|  | 35 | 6.61±0.09 c | snow white | Relatively neat | bushy | ++ |
|  | 40 | 6.51±0.11 c | white | Relatively neat | bushy | ++ |

Note:“+” poor growth; “++” growth is average; “+++” the growth is better；Different lowercase letters indicate significant differences, *p*<0.05.

## 2.2 The determination of optimal ginger straw addition ratios for the *F. filiformis* strains

Significant differences in growth rates and mycelial vigor among different strains of *F. filiformis* cultivated in ginger straw substrate at varying ratios were observed (Table S13). The J1 strain was found to exhibit a growth rate of 5.1 to 5.86 mm/d, with the highest rate of 5.86 mm/d achieved at a ginger straw addition ratio of 20%, which surpassed the growth rate of the control group. Furthermore, at ginger straw addition ratios of 20% and 25%, the growth vigor was rated as excellent, exceeding that of the control group.

The J5 strain was shown to exhibit a growth rate of 4.95 to 5.55 mm/d, with the highest rate observed in the control group, followed by substrate with ginger straw addition ratios of 15% and 20%. Mycelial vigor was found to vary across different ratios of ginger straw, with results indicating that at addition ratios of 20% and 25%, the growth vigor was rated as excellent, consistent with that of the control group.

The J7 strain was shown to demonstrate a growth rate of 5.22 to 5.74 mm/d, with the highest growth rate of 5.74 mm/d achieved at a ginger straw addition ratio of 20%, while the lowest growth rate of 5.22 mm/d was recorded in the control group. Evaluation of growth vigor indicated that at ginger straw addition ratios of 10%, 15%, and 25%, the growth vigor was rated as excellent, surpassing that of the control group.

Table S13 The growth performance of *Flammulina filiformis* strains in different proportion of ginger straw substrate.

| Strains | Addition ratio  (%) | Growth rate（mm/d） | Color | Marginal regularity | Density | Growth |
| --- | --- | --- | --- | --- | --- | --- |
| J1 | 0 | 5.1±0.06 e | snow white | Relatively neat | bushy | ++ |
|  | 10 | 5.63±0.16 abc | snow white | Untidy | bushy | ++ |
|  | 15 | 5.69±0.07 abc | snow white | neat | bushy | ++ |
|  | 20 | 5.86±0.12 abc | snow white | neat | bushy | +++ |
|  | 25 | 5.7±0.13 abc | snow white | neat | bushy | +++ |
|  | 30 | 5.4±0.07 bcd | snow white | neat | bushy | ++ |
|  | 35 | 5.4±0.11 cd | snow white | neat | bushy | ++ |
|  | 40 | 5.16±0.12 de | snow white | neat | bushy | ++ |
| J5 | 0 | 5.55±0.09 a | snow white | Untidy | bushy | +++ |
|  | 10 | 5.4±0.01 abc | snow white | Relatively neat | bushy | ++ |
|  | 15 | 5.48±.09 abc | snow white | Relatively neat | bushy | ++ |
|  | 20 | 5.48±0.19 abc | snow white | neat | bushy | +++ |
|  | 25 | 5.19±0.13 bcd | snow white | neat | bushy | +++ |
|  | 30 | 5.16±0.14 cd | snow white | neat | bushy | ++ |
|  | 35 | 5.14±0.15 d | snow white | Relatively neat | bushy | ++ |
|  | 40 | 4.95±0.14 d | snow white | Relatively neat | bushy | + |
| J7 | 0 | 5.22±0.09 cd | snow white | neat | bushy | ++ |
|  | 10 | 5.6±0.12 ab | snow white | Relatively neat | bushy | +++ |
|  | 15 | 5.56±0.11 ab | snow white | Untidy | bushy | +++ |
|  | 20 | 5.74±0.18 ab | snow white | neat | bushy | +++ |
|  | 25 | 5.39±0.04 bc | snow white | neat | bushy | ++ |
|  | 30 | 5.44±0.1 bc | snow white | neat | bushy | ++ |
|  | 35 | 5.42±0.11 bc | snow white | Relatively neat | bushy | ++ |
|  | 40 | 5.25±0.11 c | snow white | Relatively neat | bushy | ++ |

Note:“+” poor growth; “++” growth is average; “+++” the growth is better；Different lowercase letters indicate significant differences, *p*<0.05.

## 2.3 The determination of optimal ginger straw addition ratios for the *P. eryngii* strains

Variations in growth rates and mycelial vigor among different strains of *P. eryngii* were observed when cultivated in ginger straw substrate at varying ratios (Table S14). The fastest growth rate of 5.66 mm/d was recorded for the XB2 strain at a ginger straw addition ratio of 10%, significantly exceeding the growth rate of the control group. Furthermore, at ginger straw addition ratios of 10%, 15%, and 20%, the growth vigor was rated as excellent, surpassing that of the control group.

A growth rate ranging from 4.62 to 5.29 mm/d was exhibited by the XB3 strain, with the slowest growth being observed in the control group and the highest rate achieved at a ginger straw addition ratio of 25%. Moreover, at ginger straw addition ratios of 20% and 25%, the growth vigor was rated as excellent, exceeding that of the control group.

A growth rate ranging from 4.66 to 5.28 mm/d was exhibited by the XB4 strain, with faster growth than the control group being observed at ginger straw addition ratios of 10%, 15%, 20%, 25%, 30%, and 40%. At ginger straw addition ratios of 20%, 25%, and 30%, the growth vigor was rated as excellent, exceeding that of the control group.

The XB5 strain was recorded to display the slowest growth rate in the control group, at only 4.46 mm/d, while the fastest growth rate of 5.24 mm/d was achieved at a ginger straw addition ratio of 25%. Furthermore, at ginger straw addition ratios of 10%, 25%, and 35%, the growth vigor was rated as excellent, surpassing that of the control group.

Table S14 The growth performance of *Flammulina filiformis* strains in different proportion of ginger straw substrate.

| Strains | Addition ratio (%) | Growth rate（mm/d） | Color | Marginal regularity | Density | Growth |
| --- | --- | --- | --- | --- | --- | --- |
| XB2 | 0 | 5.02±0.11 c | snow white | Relatively neat | bushy | ++ |
|  | 10 | 5.66±0.09 a | snow white | neat | bushy | +++ |
|  | 15 | 5.6±0.05 ab | snow white | neat | bushy | +++ |
|  | 20 | 5.34±0.35 b | snow white | neat | bushy | +++ |
|  | 25 | 4.73±0.05 cd | snow white | Relatively neat | bushy | ++ |
|  | 30 | 4.83±0.05 cd | snow white | Relatively neat | bushy | ++ |
|  | 35 | 4.78±0.05 cd | snow white | Relatively neat | bushy | ++ |
|  | 40 | 4.56±0.04 d | snow white | Untidy | bushy | ++ |
| XB3 | 0 | 4.62±0.12 d | snow white | Untidy | bushy | ++ |
|  | 10 | 5.1±0.07 bc | snow white | Relatively neat | bushy | ++ |
|  | 15 | 4.99±0.07 bc | snow white | Relatively neat | bushy | ++ |
|  | 20 | 5.11±0.11 abc | snow white | neat | bushy | +++ |
|  | 25 | 5.29±0.07 abc | snow white | neat | bushy | +++ |
|  | 30 | 5.02±0.09 bc | snow white | Relatively neat | bushy | ++ |
|  | 35 | 4.87±0.11 c | snow white | Relatively neat | bushy | ++ |
|  | 40 | 4.93±0.12 bc | snow white | Relatively neat | bushy | +++ |
| XB4 | 0 | 4.77±0.01 cd | snow white | Relatively neat | bushy | ++ |
|  | 10 | 4.97±0.07 bc | snow white | Untidy | bushy | ++ |
|  | 15 | 4.89±0.09 cd | snow white | Untidy | bushy | ++ |
|  | 20 | 4.99±0.07 bc | snow white | neat | bushy | +++ |
|  | 25 | 5.19±0.11 ab | snow white | neat | bushy | +++ |
|  | 30 | 5.28±0.15 ab | snow white | neat | bushy | +++ |
|  | 35 | 4.75±0.06 cd | snow white | Relatively neat | bushy | ++ |
|  | 40 | 4.66±0.26 d | snow white | Relatively neat | bushy | ++ |
| XB5 | 0 | 4.46±0.2 d | snow white | Relatively neat | bushy | ++ |
|  | 10 | 4.91±0.02 c | snow white | neat | bushy | +++ |
|  | 15 | 4.93±0.09 bc | snow white | Relatively neat | bushy | ++ |
|  | 20 | 5.06±0.25 abc | snow white | Relatively neat | bushy | ++ |
|  | 25 | 5.24±0.15 abc | snow white | neat | bushy | +++ |
|  | 30 | 5.11±0.15 abc | snow white | Relatively neat | bushy | ++ |
|  | 35 | 5.31±0.06 abc | snow white | neat | bushy | +++ |
|  | 40 | 4.56±0.26 d | snow white | Untidy | bushy | ++ |

Note:“+” poor growth; “++” growth is average; “+++” the growth is better；Different lowercase letters indicate significant differences, *p*<0.05.

## 2.4 The determination of optimal ginger straw addition ratios for the *A. heimuer* strains

Significant variations in growth rates and vigor are exhibited by different strains of *A. heimuer* when cultivated in ginger straw substrate at varying ratios (Table S15). Significant differences in growth rates were shown by the h2 strain across different substrate, with the fastest growth rate of 3.72 mm/d being achieved in the control group, and the slowest growth rate of 2.29 mm/d being observed at a ginger straw addition ratio of 40%. At a ginger straw addition ratio of 20%, the growth vigor was rated as excellent, aligning with that of the control group.

A growth rate ranging from 2.44 to 3.98 mm/d was exhibited by the h4 strain, with the fastest growth being observed at a ginger straw addition ratio of 15%, surpassing the growth rate of the control group.

The fastest growth rate of 3.77 mm/d was recorded for the h7 strain in the control group, while the slowest growth rate of 2.31 mm/d was observed at a ginger straw addition ratio of 40%. The growth vigor was rated as excellent at a ginger straw addition ratio of 20%, aligning with that of the control group.

Table S15 The growth performance of *Auricularia heimuer* strains in different proportion of ginger straw substrate.

| Strains | Addition ratio (%) | Growth rate（mm/d） | Color | Marginal regularity | Density | Growth |
| --- | --- | --- | --- | --- | --- | --- |
| h2 | 0 | 3.72±0.06 a | Snow white | neat | bushy | +++ |
|  | 10 | 3.61±0.08 ab | snow white | Untidy | bushy | ++ |
|  | 15 | 3.56±0.08 b | snow white | neat | Denser | ++ |
|  | 20 | 3.51±0.03 b | snow white | neat | bushy | +++ |
|  | 25 | 3.09±0.05 c | snow white | neat | bushy | ++ |
|  | 30 | 2.59±0.04 d | snow white | Relatively neat | bushy | ++ |
|  | 35 | 2.51±0.03 d | snow white | Relatively neat | bushy | ++ |
|  | 40 | 2.29±0.1 e | snow white | Relatively neat | Denser | + |
| h4 | 0 | 3.97±0.16 a | snow white | neat | bushy | +++ |
|  | 10 | 3.76±0.04 a | snow white | Relatively neat | bushy | ++ |
|  | 15 | 3.98±0.03 a | snow white | neat | bushy | +++ |
|  | 20 | 3.78±0.03 a | snow white | neat | bushy | +++ |
|  | 25 | 3.31±0.17 bc | snow white | Relatively neat | bushy | ++ |
|  | 30 | 3.1±0.19 bc | snow white | Untidy | bushy | ++ |
|  | 35 | 2.99±0.12 c | snow white | neat | Denser | ++ |
|  | 40 | 2.44±0.14 d | snow white | Untidy | Denser | + |
| h7 | 0 | 3.77±0.05 a | snow white | neat | bushy | +++ |
|  | 10 | 3.64±0.05 b | snow white | neat | Denser | ++ |
|  | 15 | 3.61±0.01 b | snow white | Relatively neat | bushy | ++ |
|  | 20 | 3.69±0.04 ab | snow white | neat | bushy | +++ |
|  | 25 | 2.86±0.03 c | snow white | Relatively neat | bushy | ++ |
|  | 30 | 2.89±0.05 c | snow white | Relatively neat | bushy | ++ |
|  | 35 | 2.57±0.11 d | snow white | Relatively neat | Denser | + |
|  | 40 | 2.31±0.04 e | snow white | Relatively neat | Denser | + |

Note:“+” poor growth; “++” growth is average; “+++” the growth is better；Different lowercase letters indicate significant differences, *p*<0.05.

## 2.5 The determination of optimal ginger straw addition ratios for the *A. cornea* strains

Significant differences in growth rate and vigor are exhibited by different strains of *A. cornea* when cultivated in ginger straw substrate at different proportions (Table S16). The growth rate of strain M2 is found to range from 2.84 to 3.37 mm/d, surpassing the mycelial growth rate of the control group at ginger straw addition ratios of 10%, 15%, 20%, and 35%.At a ginger straw addition ratio of 15%, its growth vigor is rated as excellent, aligning with the control group.

A mycelial growth rate of 2.68 to 3.18 mm/d is exhibited by strain M6, exceeding the mycelial growth rate of the control group at ginger straw addition ratios of 10%, 15%, 25%, 30%, and 35%. At ginger straw addition ratios of 30% and 35%, its mycelial growth vigor is likewise rated as excellent, surpassing that of the control group.

Table S16 The growth performance of *Auricularia cornea* strains in different proportion of ginger straw substrate.

| Strains | Addition ratio (%) | Growth rate（mm/d） | Color | Marginal regularity | Density | Growth |
| --- | --- | --- | --- | --- | --- | --- |
| M2 | 0 | 2.94±0.09 cd | snow white | neat | bushy | +++ |
|  | 10 | 3.01±0.08 bcd | snow white | Relatively neat | bushy | ++ |
|  | 15 | 3.37±0.08 a | snow white | neat | bushy | +++ |
|  | 20 | 3.16±0.14 b | snow white | Relatively neat | bushy | ++ |
|  | 25 | 2.84±0.05 d | snow white | Relatively neat | bushy | ++ |
|  | 30 | 2.89±0.04 cd | snow white | Relatively neat | bushy | ++ |
|  | 35 | 3.05±0.07 bc | snow white | Relatively neat | bushy | ++ |
|  | 40 | 2.89±0.03 cd | snow white | Untidy | bushy | ++ |
| M6 | 0 | 2.95±0.06 c | snow white | Relatively neat | bushy | ++ |
|  | 10 | 2.98±0.05 bc | snow white | Untidy | bushy | ++ |
|  | 15 | 3.12±0.02 ab | snow white | Untidy | bushy | ++ |
|  | 20 | 2.94±0.06 abc | white | Untidy | bushy | ++ |
|  | 25 | 2.99±0.1 bc | white | Untidy | bushy | ++ |
|  | 30 | 3.18±0.1 a | white | neat | bushy | +++ |
|  | 35 | 3.03±0.11 ab | white | neat | bushy | +++ |
|  | 40 | 2.68±0.07 d | white | Relatively neat | bushy | ++ |

Note:“+” poor growth; “++” growth is average; “+++” the growth is better；Different lowercase letters indicate significant differences, p<0.05.

The optimal ginger straw addition ratios for the strains P1, P3, P4, P6, and P7 of *P. ostreatus* were determined to be 25%, 35%, 35%, 35%, and 30%, respectively. The strains J1, J5, and J7 of *F. filiformis* had optimal ginger straw addition ratios of 25%, 25%, and 20%, respectively. Optimal ginger straw addition ratios for strains XB2, XB3, XB4, and XB5 of *P. eryngii* were 20%, 25%, 30%, and 35%, respectively. The proportion of ginger straw addition in h2, h4 and h7 strains of *A. heimuer* was 20%. Strains M2 and M6 of *A. cornea* had optimal ginger straw addition ratios of 15% and 35%, respectively.
